# Chromosome Fragile Sites in *Arabidopsis* Harbor Matrix Attachment Regions That May Be Associated with Ancestral Chromosome Rearrangement Events

**DOI:** 10.1371/journal.pgen.1003136

**Published:** 2012-12-20

**Authors:** Joelle S. dela Paz, Patti E. Stronghill, Scott J. Douglas, Sandy Saravia, Clare A. Hasenkampf, C. Daniel Riggs

**Affiliations:** Department of Biological Sciences and Centre for the Analysis of Genome Evolution and Function, University of Toronto, Toronto, Canada; The University of North Carolina at Chapel Hill, United States of America

## Abstract

Mutations in the *BREVIPEDICELLUS* (*BP*) gene of *Arabidopsis thaliana* condition a pleiotropic phenotype featuring defects in internode elongation, the homeotic conversion of internode to node tissue, and downward pointing flowers and pedicels. We have characterized five mutant alleles of *BP*, generated by EMS, fast neutrons, x-rays, and aberrant T–DNA insertion events. Curiously, all of these mutagens resulted in large deletions that range from 140 kbp to over 900 kbp just south of the centromere of chromosome 4. The breakpoints of these mutants were identified by employing inverse PCR and DNA sequencing. The south breakpoints of all alleles cluster in BAC T12G13, while the north breakpoint locations are scattered. With the exception of a microhomology at the *bp-5* breakpoint, there is no homology in the junction regions, suggesting that double-stranded breaks are repaired via non-homologous end joining. Southwestern blotting demonstrated the presence of nuclear matrix binding sites in the south breakpoint cluster (SBC), which is A/T rich and possesses a variety of repeat sequences. In situ hybridization on pachytene chromosome spreads complemented the molecular analyses and revealed heretofore unrecognized structural variation between the Columbia and Landsberg *erecta* genomes. Data mining was employed to localize other large deletions around the *HY4* locus to the SBC region and to show that chromatin modifications in the region shift from a heterochromatic to euchromatic profile. Comparisons between the *BP/HY4* regions of *A. lyrata* and *A. thaliana* revealed that several chromosome rearrangement events have occurred during the evolution of these two genomes. Collectively, the features of the region are strikingly similar to the features of characterized metazoan chromosome fragile sites, some of which are associated with karyotype evolution.

## Author Summary

Chromosome evolution involves both small-scale (e.g. single nucleotide) changes, as well as large-scale rearrangements such as inversions, translocations, and fusion events. We investigated mutations of the *BREVIPEDICELLUS* gene of *Arabidopsis*, which is a master regulator of inflorescence architecture. These mutations are not due to single nucleotide changes, but rather to large deletions, some spanning nearly one million base pairs. Molecular and biochemical analyses reveal that the chromosome breakpoints cluster in an area that is rich in repetitive elements and harbor multiple binding sites for nuclear matrix proteins. Data mining revealed intriguing correlations between the breakpoint cluster and hotspots of genetic recombination, regions of the chromosome that have undergone several rearrangement events during evolution, and changes in histone protein modifications. We propose that these unstable regions are chromosome fragile sites that assist in marking a boundary between gene-poor, transcriptionally repressed centromeric chromatin and a more relaxed chromatin domain that is gene-rich.

## Introduction

Genome integrity depends upon the coordination of replicon and centriole duplication, chromatin condensation, and the assembly and action of the spindle apparatus. Several checkpoints regulate the progression of the chromosomal and cytoskeletal events [1], and repair systems are recruited as needed to correct replication errors and lesions caused by intrinsic and extrinsic mutagens. Intrinsic mutations may result from the interaction of DNA with reactive metabolites (e.g. hydroxyl radicals) and through the activation of mobile genetic elements. Forward genetics proceeds by employing mutagens, which can range from simple chemical mutagens such as ethyl methanesulfonate (EMS), that typically induce base substitutions, to insertional mutagens such as viral and T-DNA integration, to ionizing radiation that is often associated with rearrangements and/or deletions. Characterization of naturally occurring and induced mutants has dramatically accelerated our understanding of all cellular processes and has led to the discovery of small regulatory RNA molecules and epigenetic modifications of chromatin, two areas of intense investigation in contemporary biology.

The eukaryotic nucleus is organized hierarchically. The basic level of chromatin organization centers on the nucleosome and through various levels of organization and compaction, nucleosome strings are organized into large loop domains that are anchored to the nuclear matrix [2]. Superimposed on this, specific chromosome boundaries or territories exist within the nucleus, further defining the association of specific inter- and intrachromosomal domains [3]. Double strand breaks (DSB), created in the context of recombination activities, or due to mutagen exposure, must be repaired to ensure chromosome integrity. The juxtapositioning of specific chromosomes likely underpins the recurrent nature of specific rearrangements, for example the reciprocal translocation between human chromosomes 9 and 22 associated with chronic myelogenous leukemia.

The evolution of chromosomes has been intensively studied in many systems and is being revolutionized by high throughput sequencing technologies. In plants, genome duplication followed by translocations, inversions, centromere shifts, and the activity of endogenous mobile elements are deemed to be responsible for dispersed blocks of synteny that are observed between distantly related species [4], [5]. The breakpoints for some of these rearrangements have now been mapped onto reference genomes, and this has facilitated a high-resolution comparison between *Arabidopsis thaliana* and *A. lyrata* [6], which are believed to have evolved from a common ancestor. A series of chromosome fusion and other rearrangement events, coupled with DNA loss, has reduced the chromosome number from eight to five and the amount of DNA by approximately 40%. Within the *Brassicaceae*, it is clear that 24 conserved chromosomal blocks have been rearranged during evolution to constitute the genomes of mustard family members [7]. At present, it is unknown whether structures at the boundaries of the blocks promote recombination/rearrangement events or if repressive structures exist elsewhere to maintain the syntenic blocks.

We previously reported that the *brevipedicellus* phenotype of Arabidopsis is due to loss-of function of the *KNAT1* homeodomain protein-encoding gene and that unusually large deletions occur with a high frequency [8], [9]. Here we report the characterization of the breakpoint junctions of five *bp* deletion alleles and the conservation of their features with metazoan chromosome fragile sites, some of which are associated with chromosome breakage events that occurred during the evolution of eukaryotic karyotypes.

## Results

### Delineation of Deletion Breakpoints and Analyses of Sequence Junctions

Our previous work documented that five different *brevipedicellus* alleles were not simple alterations in the gene sequence, but rather were due to large deletions of at least 150 kbp [8]. Information on all reported *bp* alleles can be found in Table S1. The original *bp-1* mutant, isolated by Koornneef and coworkers [10], was generated by EMS mutagenesis, an alkylating agent that typically induces G to A transition mutations. We identified the *bp-2* and *bp-3* mutants from a fast neutron mutagenized population; *bp-5* is the result of an aberrant T-DNA insertion, and *bp-11* is an x-ray induced mutant. All five of these alleles exhibit large deletions. The other characterized *bp* mutants appear to be simple base changes induced by EMS (*bp-4, 6–8, and 10*), or are insertional mutants (*bp-9*). Lastly, Venglat et al. [11] reported the isolation of a *bp-2* mutant from a promoter tagged population; this mutant was later characterized as a point mutation.

**Figure 1:**
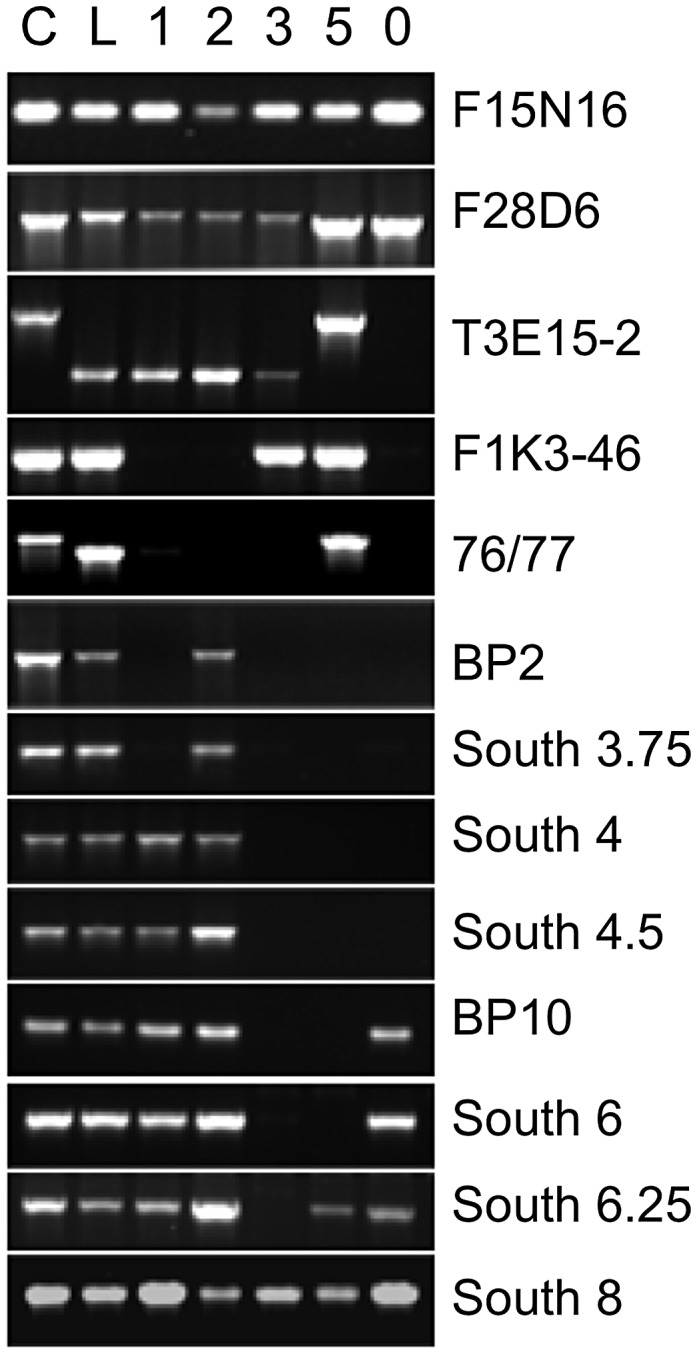
Localization of *bp* allele breakpoints by PCR. DNA from Columbia (C), Landsberg *erecta* (L), and *bp* alleles, *bp-1*, *2*, *3*, *5* and *11* (designated 0) were amplified with primer sets spanning approximately 1.2 Mbp of chromosome 4. Note that L*er* DNA is polymorphic for some loci, leading to either no amplification or to products of a different size than found for Columbia (AFLPs). For the *bp* alleles the absence of a PCR product is interpreted as representing a deletion of that region of the genome. Primer information and data for additional primer sets that punctuate those shown in the figure can be consulted in Table S2.

Delineation of the boundaries of these deletion alleles would permit an analysis of breakpoint regions and perhaps provide clues as to why the region appears to be prone to such extreme segmental deletion events. We therefore employed a six-phase strategy to determine the breakpoints of the five deletions, followed by in-silico analyses to search for motifs that might be important determinants of either generating the deletion or limiting the extent of the lesion. First, for each allele, at least one breakpoint was generally localized by employing PCR, using sets of primers that span a region of approximately 1.2 Mbp. The absence of a PCR amplification product was interpreted to mean that the region was part of the deletion (Figure 1). Next, DNA gel blotting was employed to find a restriction fragment length polymorphism (RFLP) between the mutant and parental (either Columbia or L*er*) DNA. In phase 3, mutant DNA was cleaved with the enzyme identified by RFLP analysis and the DNA was ligated under conditions designed to produce intramolecular events, generating circular products. This DNA was used as a template for inverse PCR (iPCR) to amplify sequences adjacent to known DNA, including the repaired breakpoints. DNA sequencing was then employed to determine the breakpoint junction sequences. Based on this information, new primers were designed to amplify mutant DNA across the breakpoints to give rise to products of predicted size and sequence and thus validate the deletion (phase 5 analysis). Lastly, computer algorithms were employed to analyze the breakpoint junctions, to search for common features/motifs.

Table 1 summarizes the five *bp* deletion alleles. Sequencing of iPCR products revealed that both *bp-1* and *bp-2* north junction sequences are composed of highly repeated sequences that are found predominantly in centromeric regions on all five chromosomes. For *bp-1*, the breakpoint occurs within a tandem (AG)^n^ motif that is broadly distributed and may be a member of the LIMPET transposon family [12]. The north junction sequence could not be unequivocally localized for the following reasons. First, we found that a substantial number of primer sets, spanning BACs F5K24, F10A2, T3E15, and F28D6, did not give rise to PCR products with Landsberg *erecta* DNA templates (and therefore were PCR negative for all *bp* alleles in an L*er* background; see Tables S2, S3, S4 for primer sequences, locations and amplicon status). Other primer sets generated AFLPs wherein the PCR product size differed between L*er* and Col, but which can be useful tools for map-based cloning (e.g. primer sets 76/77 and T3E15-2 in Figure 1). Second, sequencing of iPCR products revealed that the junction sequence adjacent to the south *bp-1* breakpoint is most similar to sequences found on other chromosomes, particularly chromosomes 1 and 3. Intriguingly, the percent match for all of these sequences is only 80–91%. The best matches on chromosome 4 include a sequence near 1 Mb, which could be interpreted as an inversion involving the centromere. Additionally there is homology to a region centered at about 4.3 Mb that has a polarity opposite that expected from sequencing the iPCR product. Subsequent pachytene chromosome in situ hybridization discounted the first possibility as cytological landmarks and the distances between fluorescent probe signals are normally distributed in *bp-1* (see below). Although a complex rearrangement may be involved, it is more likely that sequence divergence between the Columbia and Landsberg *erecta* genomes is responsible for this disparity. Based on the locations of the primer sets, *bp-1* has suffered a deletion of at least 400.6 kbp (south breakpoint to primer set F1K3) and possibly as much as 900 kbp (south breakpoint to homologous region at 4.3 Mb).

**Table 1:** Summary of *bp* deletion alleles.

Allele	Mutagen	Genetic background	North breakpoint	South breakpoint	Deletion size
*bp-1*	EMS	L*er*	ND	5.203	400–900 Kbp
*bp-2*	FN	L*er*	ND	5.165	>850 Kbp
*bp-3*	FN	L*er*	4.861	5.248	386,634 bp
*bp-5*	T-DNA	Col	5.107	5.243	140,996 bp
*bp-11*	X-rays	Col	4.288	5.212	925,158 bp

ND = not determined.

Mutagens: EMS: ethyl methanesulfonate; FN: fast neutrons.

Breakpoint locations are expressed in megabase pairs.

The *bp-2* south breakpoint is located 13.7 kbp south of the *BP* gene, within the third intron of At4g01870, encoding a putative IP3 kinase. The sequence north of this breakpoint exhibits significant homology to a centromeric satellite sequence that is highly repeated on all chromosomes. The best match on chromosome 4 is to BAC F13J5 (94%), followed by the adjacent BAC, F15N16 (93%). It is noteworthy that the *bp-2* allele, also in the L*er* background, is PCR positive for three primer sets within BACs T3E15, F28D6 and F14G16, all of which are south of F15N16 according to the AGI reference sequence. The simplest explanation of these data is that either Columbia or L*er* has suffered an inversion that positions the F13J5 and F15N16 sequences closer to the *BP* locus. Additionally or alternatively, sequence divergence between the centromeric satellites in Col vs. L*er* might account for the less than perfect sequence homology we found. As is the case for *bp-1*, the repetitive nature of the *bp-2* flanking sequence prohibits a phase 5 PCR analysis to amplify across the breakpoints and thus the extent of the *bp-2* deletion cannot be unequivocally known. However, with the Columbia BAC tiling path as a reference, the deletion in *bp-2* is at least 851 kbp based on the localized south breakpoint at AGI coordinate 5164664, and the first PCR positive fragment on the north end (in BAC T3E15 centering at 4.3 Mbp).

The locations of both junctions for *bp-3*, *bp-5* and *bp-11* were unequivocally determined. *bp-3*, another fast neutron generated allele, suffered a precise deletion of 386,634 bp, with a single guanosine residue inserted between the two breakpoints. *bp-5*, which presumably arose due to an aberrant T-DNA integration event, suffered a 140,996 bp deletion, but possesses a 19 bp ‘filler’ sequence (5′ TCCATGTAGTAAGGTAATT3′) at the junction that is 90% identical to a sequence on chromosome 3. The phase 5 PCR product sequence validates the deletion boundaries and the foreign insertion sequence, but its origin is unknown. Lastly, *bp-11* is an x-ray induced allele in which the precise excision of 925,158 bp occurred.

### FISH Analyses Confirm Cytological Landmarks and Reveal Large-Scale Ecotype-Specific Chromosome Polymorphisms

**Figure 2:**
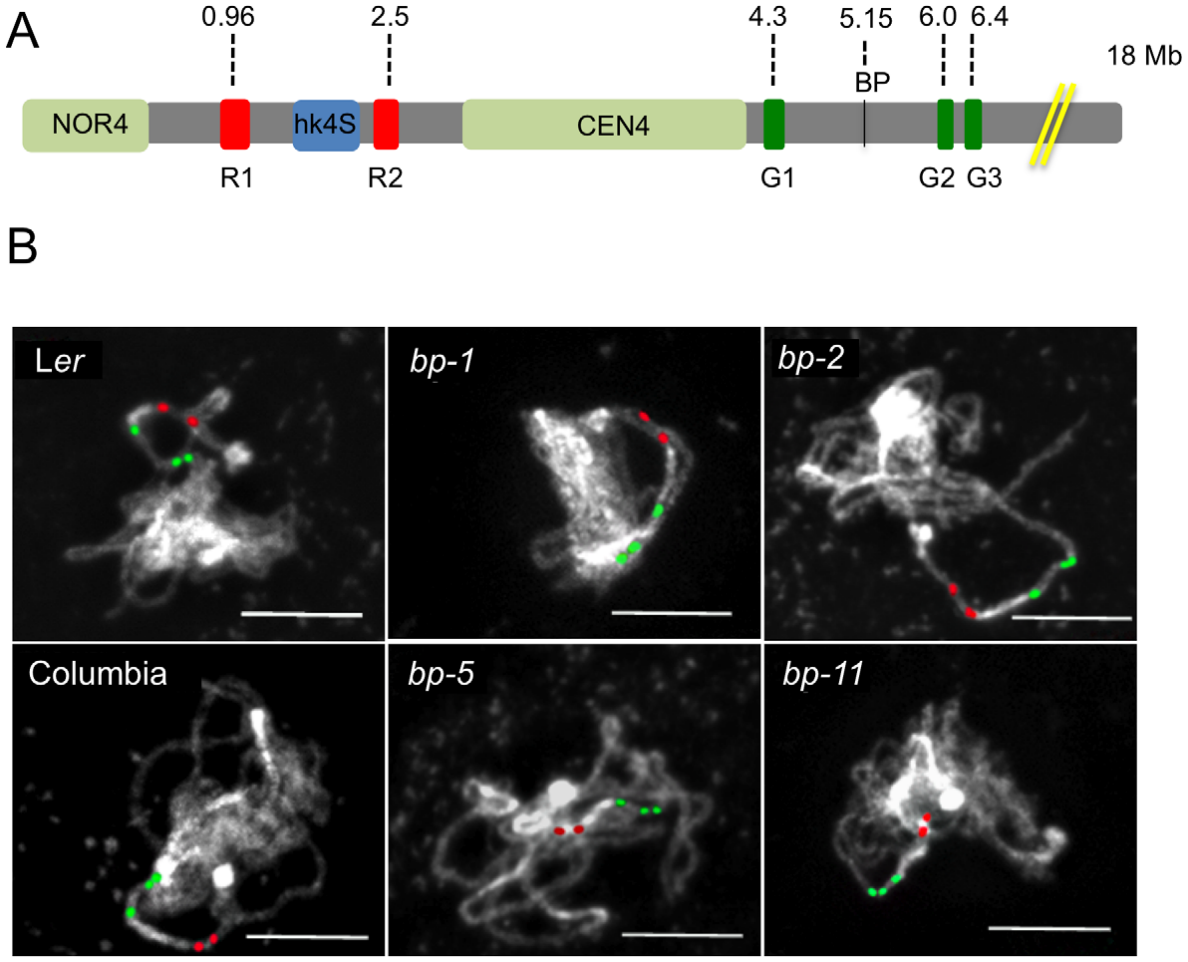
Chromosomal landscapes for Columbia, Landsberg *erecta*, and the *bp* alleles reveal ecotype specific and allele specific differences. A. Cytological map of the north end of chromosome 4 showing the nucleolar organizer (NOR4), the heterochromatic knob that exists in Columbia (hk4S), and the centromere region (CEN4). The locations of the *BP* gene and the red and green in situ hybridization probes and their AGI coordinates are shown. The figure is drawn to scale, with the exceptions that NOR4 and CEN4 are only allotted 1000 bp in the AGI numbering scheme. Cytologically, these are large regions, occupying 2–4 Mbp and have been represented as such to enable comparisons of DAPI stained landmarks vs. FISH signals. B. Representative pachytene in situ chromosome hybridization patterns for the samples listed. Scale bars represent 10 microns.

To provide complementary data on the breakpoint locations determined by our molecular analyses, and to investigate the extent of the deletions in *bp-1* and *bp-2*, we coupled cytological analyses with pachytene chromosome fluorescence in situ hybridization (FISH), using five probesets that span chromosome 4 from 0.7 Mbp to 6.4 Mbp (Figure 2). In Columbia, an inversion event involving pericentric heterochromatin has resulted in a heterochromatic knob in this ecotype, which has no counterpart in L*er* [13], [14]. Cytological examination of DAPI stained chromosomes enabled us to measure distances between the NOR4 ribosomal gene cluster at the north end and other cytological landmarks: the heterochromatic knob (hk4S, in Columbia backgrounds), and CEN4 (Table 2). In addition, the size of hk4S and CEN4 could be evaluated. These analyses revealed that the distance from NOR4 to CEN4 and the size of CEN4 are approximately equal in both parental lines and in all *bp* alleles. In the Columbia based alleles, the size of hk4S is also similar to the wildtype parent line, as was expected.

**Table 2:** Cytological and FISH analysis of Columbia, Landsberg *erecta*, and the *bp* alleles.

GENOTYPE	NOR4 – RED1	NOR4 - CEN4	hk4S SIZE	RED1-RED2	CEN4 SIZE	RED2 –GREEN1	GREEN1 –GREEN2
WT Columbia (n = 7)	5.5±0.25	7.7±0.17	0.7±0.05	1.1±0.07	4.2±0.39	5.6±0.29	2.7±0.20
*bp-5* (n = 6)	5.5±0.07	7.8±0.42	0.8±0.05	1.1±0.00	4.2±0.29	5.6±0.49	2.1±0.23
*bp-11* (n = 12)	5.3±0.14	7.6±0.41	0.8±0.08	1.2±0.11	4.1±0.21	5.7±0.36	1.7±0.18
L*er* (n = 8)	5.4±0.15	8.3±0.27	N/A	2.4±0.23	4.2±0.20	4.9±0.15	5.2±0.27
*bp-1* (n = 15)	5.0	7.9±0.0	N/A	2.0±0.12	4.3±0.07	5.2±0.14	4.5±0.23
*bp-2* (n = 9)	5.2	8.0	N/A	2.0±0.09	4.2±0.24	5.1±0.25	4.2±0.27

All signal distances and the size of the centromere and hK4S are in microns.

N/A: not applicable as L*er* backgrounds lack hk4S.

The probesets for FISH were chosen to evaluate distances between distinct cytological landmarks and the probe target sequences. The two rhodamine-associated probesets RED1 and RED2 bracket hk4S in Columbia (Figure 2a) and measurements of NOR4 to RED1 revealed no significant differences in either of the two parental ecotypes, or in the *bp* alleles. As expected, due to the pericentric inversion in Columbia that generated hk4S, the RED1 to RED2 interval is smaller in Columbia than in L*er*, and the distances for the *bp* alleles are similar to their parent ecotypes. We conclude that the *bp*-associated deletions do not involve chromatin north of the centromere. In this regard, the possibility that the *bp-1* north junction sequence, which exhibits homology to the 1 Mbp region suggestive of an inversion involving the centromere, can be ruled out in favor of an event occurring south of the centromere in a region that has diverged between Columbia and L*er*.

To correlate the size of the deletions with cytological measurements, three additional probesets were employed. GREEN 1 sequences are located very near CEN4 at 4.3 Mbp, while two additional GREEN probes bind in the 6–6.4 Mbp region, south of the BP locus at 5.15 Mbp (Figure 2). We expected that the extent of the deletions, as gauged by molecular analyses and sequence comparisons, could be roughly correlated with changes in the distance between GREEN1 and GREEN2 (G1/G2). Indeed, this is the general trend we observed as *bp-5* (141 Kbp) and *bp-11* (925 Kbp) deletions gave intersignal distances of 2.1 µm and 1.7 µm, respectively, compared to the wildtype Columbia distance of 2.7 µm. For the L*er*-based alleles *bp-1* and *bp-2*, for which the north breakpoints could not be established, we observed similar reductions in signal distances compared to the L*er* parental background with *bp-2* exhibiting a shorter G1/G2 length, and thus a larger deletion, than *bp-1*.

To our surprise, we found that the G1/G2 distance in Columbia is markedly different from L*er*, being on average about 2.7 µm in Columbia, but over 5 µm for L*er*. These measurements were reproducible and statistically significant, implying that a major polymorphism exists between these two ecotypes. Because the RED2 to GREEN1 distances do not vary significantly between Col and L*er* and because GREEN1 localizes very close to the DAPI stained centromere in both ecotypes, it seems likely that the G1/G2 polymorphism is due to an indel occurring between them, or possibly an inversion that moved the G2/G3 probeset sequences in a manner similar to the event that created hk4S. The latter possibility might be resolved by using different colored G2/G3 probes in future experiments.

### Bioinformatic Analyses of Breakpoint Regions

Simple BLASTn searches coupled with gene annotations available through the TAIR database permit the identification of genetic elements at breakpoint regions. As the *bp* deletions are found in the pericentromeric region, there are many occurrences of transposons, pseudogenes, and repetitive sequences. Some of these may be expressed, based on annotated cDNAs and/or expressed sequence tags that map to these sequences, but due to their repetitive nature, it is not clear if they are the actively expressed copies. Discounting transposon/repeat-associated sequences, in the region of 4.2 Mbp to 5.25 Mbp, there are 31 annotation units for which cDNA/EST annotations exist, and another 16 predicted genes for which there are no cDNA/EST sequences. The largest deletion, *bp-11*, which spans over 925 kbp, has lost 27 genes, while *bp-5*, the shortest deletion allele spanning 141 kbp, is missing 10 genes (see Table S5). Within the set of 31 genes, there are two pseudogenes, nine that encode unknown proteins, and three that encode proteins with conserved domains of unknown function. Genes with more complete annotations are mostly members of gene families, though a few are single copy genes. Under normal growth conditions, the L*er* based alleles are indistinguishable from one another, and the Columbia based alleles are also similar to one another. It must be appreciated that the *bp* phenotype is enhanced in the L*er* background due to the absence of the *ERECTA* protein kinase [9]. Our initial work with *bp-2* demonstrated that the *bp* mutant phenotype can be rescued by transformation with a wildtype gene [8]; thus under normal growth conditions, the deleted genes seem to be dispensable.

**Figure 3:**
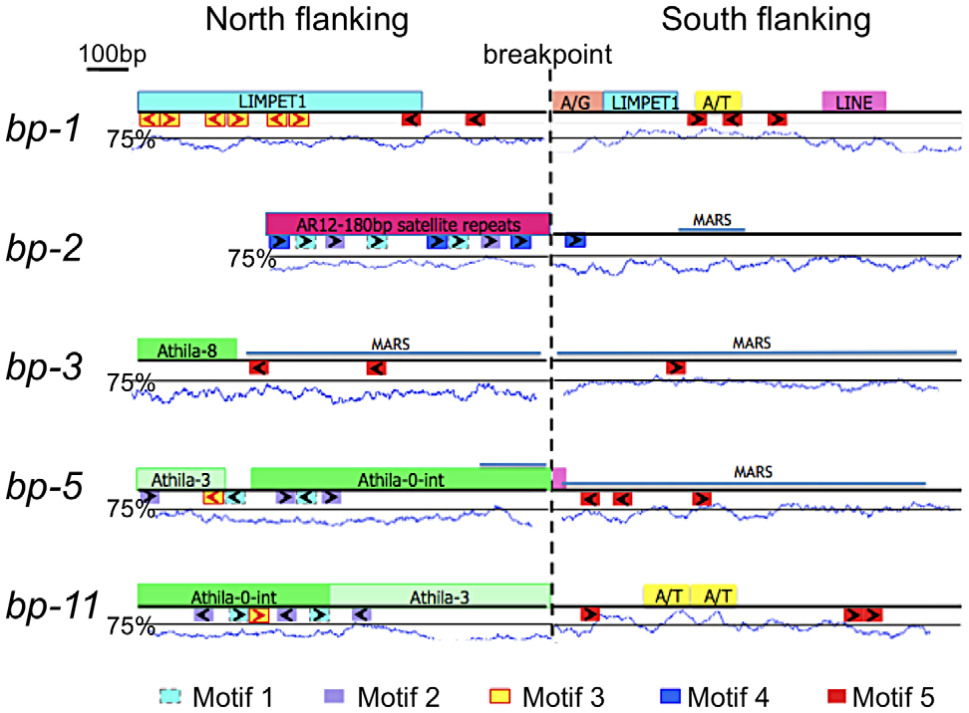
Bioinformatic analysis of *bp* alleles. One kilobase of flanking sequence north and south of the breakpoint is represented. Above each line is a summary of Repeatmasker analysis that identified common repetitive elements and low complexity regions. A/T represents an adenine/thymine rich region, while A/G represents an adenine/guanine repeat. The A/T content is graphed below the boxes with the solid line positioned at the 75% threshold for a window size of 50. Below each line is a summary of MEME analysis, which identified common sequences represented as colored boxes (motifs 1–5). The orientation of these repeats is given by the arrows within the boxes. Figure S1 shows the MEME sequence alignments and their p-values. The locations of predicted MARS binding sites are shown as labeled blue lines.

A suite of bioinformatics algorithms (BLAST, MEME, RepeatMasker) was used to interrogate the breakpoint sequences to discover commonalities that might inform our understanding of how the lesions were generated/repaired. Our strategy was to analyze north and south donor sequences. A north donor consists of 1 kbp of DNA north of the north breakpoint linked to 1 kbp of adjacent DNA that was deleted. Similarly, a south donor consists of 1 kbp of DNA that was deleted adjacent to 1 kbp of DNA south of the south breakpoints. As the breakpoint regions lie in the pericentromeric chromatin, most of the breakpoint donor sequences were found to possess one or more known repeats and/or transposon remnants (Figure 3). There is no common sequence motif shared by all alleles, but several short repeats of 20–50 nucleotides (motifs 1–5, see also Figure S1 for alignments and their p-values) are conserved in three to four alleles. The north flanking sequences are predominated by satellite (*bp-2*), Athila/gypsy transposons (*bp-3*, *bp-5*, *bp-11*) or LIMPET elements (*bp-1*), most of which are abundant and dispersed throughout the pericentromeric region. The south flanking regions, which cluster within 80 kbp of each other in BAC T12G13, tend to be sparse in repetitive sequences, but are A/T rich and 80% possess motif 5, a T-rich element that is also found in two of the north flanking regions. Statistically, the p-values for motifs 1–5 range from 2.8×10^−22^ to 5×10^−6^, lending credibility to their association with lesion formation and/or repair.

Sequences at the breakpoints offer little evidence of homologous recombination. Although both the north and south flanking regions of *bp-1* contain sequences with homology to LIMPET elements, the junctions are not a continuous sequence in the repaired product, which argues against homologous recombination. A simplistic explanation for this lesion is that it results from illegitimate recombination between (AG)^n^ rich repeat sequences located at approximately 4.2 and 5.2 Mbp, but the significance of the flanking LIMPET sequences cannot be evaluated. Additionally, the close proximity of a (CT)^n^ rich sequence juxtaposes two complementary sequences that could generate a long hairpin structure with the breakpoint occurring in a loop of four nucleotides at its apex. Complex secondary structures are predicted at or near all of the donor sequences, with the exception of *bp-1* (see Figure S2).

The apparent lack of homology between the paired north and south flanking sequences indicates that the DNA damage is likely repaired by non-homologous end joining (NHEJ). In only one instance (*bp-5*) is there microhomology between the 5′ and 3′ donor sequence junctions, which might portend the involvement of microhomology-mediated recombination.

### Breakpoint Junctions Associate with a Nuclear Matrix Protein In Vitro

**Figure 4:**
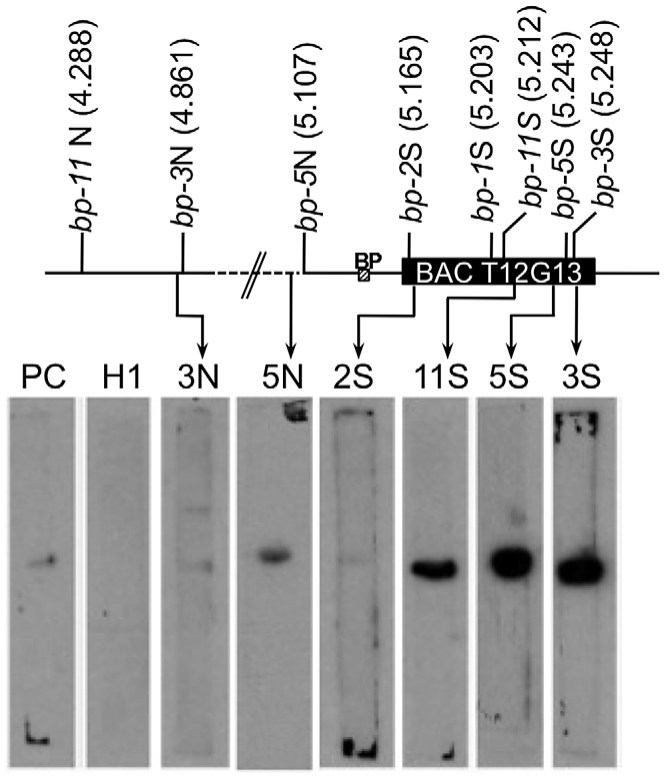
Breakpoint junction regions bind the nuclear matrix protein AHL1. Aliquots of proteins from induced *E. coli* harboring HIS-tagged AHL1 were subjected to SDS-PAGE and blotting, and membrane strips were probed with end labeled DNA fragments. 2S, 3N, 3S, 5N, 11S represent probes near the north or south (N or S) borders of the breakpoints of each *bp* allele as shown on the accompanying map with AGI coordinates in Mbp. PC represents a positive control from the plastocyanin gene [16] while H1 represents the histone H1 gene (At1g06760) probe, used as a negative control. The locations of the *BP* gene and BAC T12G13 are shown.

Matrix attachment region (MAR) prediction algorithms suggested the possibility that some of the sequences near the breakpoint junctions contain binding sites for the nuclear matrix. There are several known components of the nuclear matrix, including AHL1, an AT hook domain containing protein that has been shown by biochemical and cytological assays to be associated with the nuclear matrix [15]. We cloned an *AHL1* cDNA behind an inducible promoter and expressed it as a His-tagged protein in *E. coli*. Southwestern blot analysis was then undertaken with end-labeled probes, including a positive control (a plastocyanin gene, PC, [16]), a negative control (a histone H1 gene fragment, At1g06760) and several fragments near the breakpoint junctions (2S, 3N, 3S, 5S, 5N, and 11S). Figure 4 shows that the histone H1 probe does not bind to the AHL1 protein. The positive control PC1 probe as well as the 2S, 3N and 5N probes bound weakly, but above background levels, while the 3S, 5S and 11S probes exhibited strong binding to AHL1. We conclude that these probes, representing regions predicted to contain MAR binding sites, do indeed bind to a known matrix associated protein. Intriguingly, the 3S, 5S and 11S probes are located within 26 kbp of each other at the south end of BAC T12G13, which harbors all of the south end deletion breakpoints. It is conceivable that BAC T12G13 contains sequences that organize chromatin loops and that in the *bp* deletion mutants, resection of the initial lesions is limited by either a complex chromatin structure (e.g. boundary element possessing extensive secondary structure) and/or an attachment point on the nuclear matrix.

### Crossover Frequency and Genome Polymorphisms Are Indexed by the *Bp* Breakpoints

**Figure 5:**
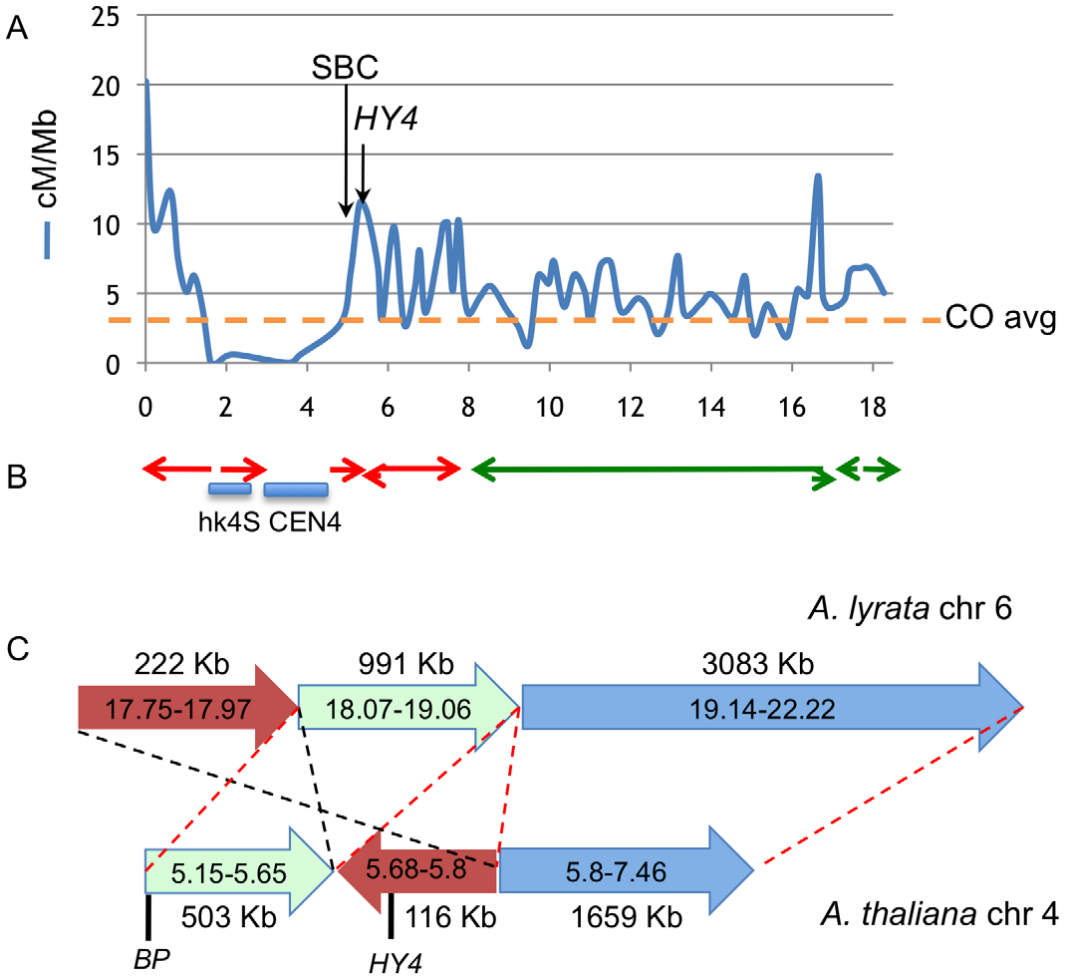
Colocalization of breakpoints with recombination hotspots and chromosome rearrangement events. A. Recombination frequency (in cM/Mbp) along chromosome 4, recharted from data of Drouaud et al [17]. The horizontal axis values are megabase pairs, with the north end of the chromosome on the left. The average recombination frequency (CO) is shown by the horizontal hatched line. B. Locations of gross chromosomal fusion and rearrangement events in Arabidopsis progenitors that gave rise to *A. lyrata* and *A. thaliana*. The locations of *A. lyrata* chromosomes 6 (red) and 7 (green) segments are depicted as the colored arrows [6]. The direction of the arrows indicates the polarity of rearranged segments. Not all fusions/inversions are shown. Note the general correlation between CO frequency, the cluster of *bp* south breakpoint deletions (SBC), and the location of chromosome rearrangements. The HY4 arrow points to a region around 5.7 Mbp where numerous *HY4* deletion mutants exist (see the Discussion and [18]). C. High resolution sequence comparison of *A. lyrata* chromosome 6 (top) and *A. thaliana* chromosome 4 (bottom) in the pericentric region where *BP* and *HY4* are located. Three *A. lyrata* genome segments are shown along with their lengths and are color coded. The corresponding *A. thaliana* regions are shorter and ancestral rearrangement events have repositioned the *BP/HY4* loci relative to the progenitor genome. The red hatched lines and segment polarity arrows indicate colinearity/synteny between the two genome segments, while the black hatched lines highlight a putative inversion/transposition event(s) involving the brown colored segments. In addition to the rearrangement events, note that significantly more DNA exists in this region in *A. lyrata* relative to *A. thaliana*, implying that additional remodeling events have also occurred. Sequences to the left of each chromosome represent pericentromeric DNA that cannot be accurately aligned due to the repetitive nature of the region.

The lack of an ordered array of large clones of L*er* DNA precludes both direct Col/L*er* synteny comparisons as well as the construction of genome array chips for chromatin immunoprecipitation analysis. Nevertheless, as two of our *bp* alleles are derived from Columbia, we suspected that data mining might prove fruitful for correlating the breakpoint regions with established chromatin features and genetic data. We reasoned that segments of the genome that are active in recombination might have features that could predispose them for rearrangement events. High-resolution recombination mapping along chromosome 4 revealed very little recombination in the hk4S and CEN4 regions, as expected, but also identified regions which exhibit high levels of recombination (hotspots, [17]). Figure 5 shows that the 5–6 Mbp region is very active in recombination, with one of the hotspots in the same region where the south breakpoints of all *bp* alleles cluster. Interestingly, this region also includes the *HY4* locus, where multiple large deletion alleles have been reported [18].

*A. thaliana* and *A. lyrata* likely evolved from a common ancestor and several chromosomal fusion and rearrangement events have occurred to reduce both the size of the genome and the number of linkage groups in *A. thaliana* [6], [7]. Comparisons of the genomic sequences of the two species reveal that several regions of the ancestral chromosomes six and seven were fused to generate *A. thaliana* chromosome 4 [6], [7 and references therein]. Importantly, some of the major rearrangement events also map to the region where the south breakpoints are clustered and in other areas, for example in the 16–17 Mbp region where other rearrangement events have occurred, recombination hotspots also exist (Figure 5B). Comparative sequence analysis of the *BP/HY4* region of the *A. thaliana* and *A. lyrata* genomes indicate that one or more segments of these genomes underwent transposition/inversion events, providing additional evidence that the region is recombinogenic and prone to chromosome rearrangement events that are possibly associated with speciation (Figure 5C).

**Figure 6:**
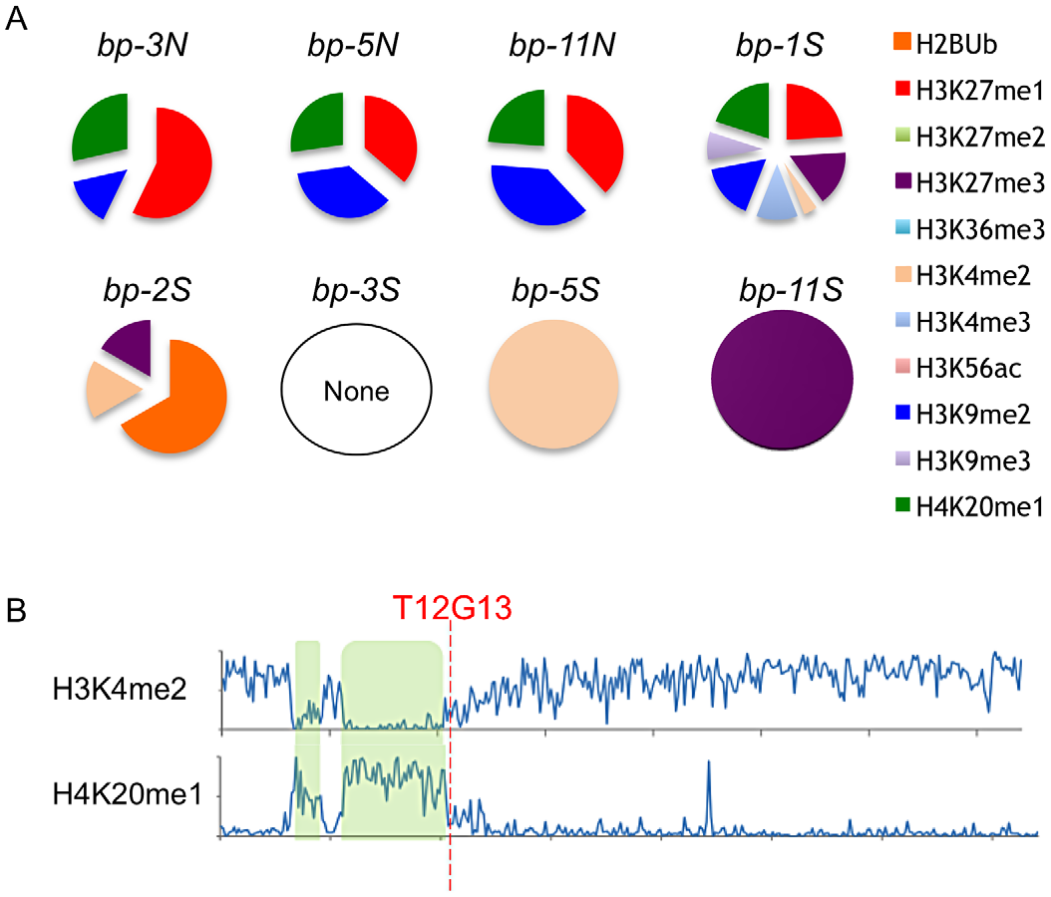
Chromatin modifications at the breakpoint junctions. A. North breakpoints occur in pericentric heterochromatin and bear H3K9me2, H4K20me1 and H3K27me1 signatures. South breakpoint modifications exhibit no epigenetic marks or euchromatic marks. B. Global view of chromosome 4 euchromatic (H3K4me2) and heterochromatic (H4K20me1) modification patterns. hk4S and CEN4 positions are highlighted in green; the position of BAC T12G13 at the transition zone is indicated (data from [19]).

Epigenomic data mining revealed that the defined north end breakpoints (*bp-3*, *bp-5* and *bp-11*) possess common histone modifications, specifically H3K27me1, H3K9me2 and H4K20me1, and in addition exhibit 5–15% 5-methylcytosine (Figure 6, see also Figure S3). This combination of chromatin modifications is associated with transposable element rich regions found in pericentric heterochromatin [19]. The south end breakpoints, with the exception of *bp-1*, differ markedly from the north ends, sharing no chromatin marks and exhibiting very low levels of 5-methylcytosine. The *bp-2* south breakpoint, located within an expressed gene, contains ubiquitinated H2B, H3K27me3 and H3K4me2, all typically associated with euchromatin [19]. The other clustered south breakpoints contain either of the two latter modifications (*bp-5*, *bp-11*), but are generally devoid of chromatin marks. However, the local regions possess a variety of modifications and the four basic chromatin states [19] are interspersed (see Figure S3). It is conceivable that a particular combination of chromatin modifications may promote genome instability, or, as we observed for the clustered south breakpoints, a dearth of chromatin modifications and lack of 5-methylcytosine may be indicative of a chromatin state that underpins genomic instability/recombinogenic potential. In any event, features inherent to BAC T12G13 represent a boundary element or transition zone in which the chromatin state switches from one bearing heterochromatic marks to one indicative of a more euchromatic state (Figure 6B).

### The *BP/HY4* Region Is Unstable Relative to Many Other Loci

The large deletions that we document for the *BP* locus, as well as the close proximity of the *HY4* locus where numerous large deletions have also been reported [18], encourages speculation that the region is generally unstable and prone to deletions and other gross chromosomal rearrangement events. We therefore examined literature reports and employed mutant germplasm search engines to catalog the locations of deletions greater than 25 bp to ascertain if the *BP/HY4* region is overrepresented in this data set. Figure S4 shows that the 5 *bp* alleles and the 15 *hy4* alleles reported by Bruggemann and coworkers [18], constitute 20 of the 120 deletion mutations associated with structural genes. We conclude that the *BP/HY4* region is on average more prone to deletion/rearrangement events than most other regions of the genome.

## Discussion

Genome evolution is driven by a variety of factors, not the least of which are gross chromosome rearrangements that are sponsored by the activity of mobile genetic elements, first reported by Barbara McClintock [20]. More recently, mechanistic analyses of structural variation in a variety of organisms have focused on errors in replication and recombination as causative [21]–[23]. Supporting evidence is provided by the existence of sequences with homology to transposons and/or repeat motifs at breakpoint junctions, evoking homologous recombination or microhomology mediated non-homologous end joining (NHEJ) mechanisms for lesion repair. In the absence of evidence for homologous recombination and outside of the context of meiosis, NHEJ is a pathway to repair [24], and ligation of free ends is a stochastic process, often resulting in translocations. The pericentric chromatin in which *BP* resides would seem to be inherently unstable due to the presence of autonomous and non-autonomous transposons of various types, numerous repetitive sequences, and sequences of unusual base composition that may impose constraints on replication fidelity due to the formation of secondary structures.

In instances where mutagenesis is employed and which result in gross chromosomal rearrangements, it is not clear if the initial event is associated with DSB formation followed by resection of the free ends and final repair, or alternatively, if there are two DSBs that upon repair result in loss of the intervening sequence. With respect to the *bp* deletions, it is intriguing that several different mutagenic agents (EMS, fast neutrons, T-DNA insertion and x-rays) produce the same effect: large deletions. Four of the five *bp* alleles possess Athila LTR retrotransposon elements at or near the north breakpoint junction and the fifth contains satellite repeats at the junction. As there is no corresponding transposon/satellite DNA south of the breakpoint, there is little evidence to support transposon movement, homology-based recombination or replication slippage. An exception is *bp-5*, where the microhomology found at the junction could suggest one of the latter two processes may have been involved. In the absence of evidence for these types of events in the other alleles, we speculate that the initial break likely occurred during DNA replication, and that resection, likely mediated by the MRN complex [25], extended the region. The extent of DNA loss could be limited by either a change in the structure of DNA (e.g. alternating dinucleotides or elaborate secondary structures, as is observed for several *bp* alleles), a change in the chromatin microenvironment (e.g. accessibility changes due to differences in histone modification patterns and binding by their associated proteins), and/or a repressive influence of protein factors (e.g. encountering a nuclear matrix protein complex as we report for the south breakpoint cluster).

### Breakpoint Junction Sequences Exhibit Features of Metazoan Fragile Sites

Chromosome fragile sites have long been recognized in metazoans as regions which are prone to breakage when replication stress occurs, and activation (breakage) of some are implicated in various disease states and cancer [26], [27]. Some of the common fragile sites that have been characterized at the molecular level are associated with breakpoints that delineate homologous syntenic blocks that have persisted during the evolution of eukaryote karyotypes [28]. Although a wealth of data correlates recurrent deletions and rearrangements with these fragile sites, very little is known about the initial breakage events, but rather only the sequences of junction regions provide clues as to the repair pathway. Some fragile site breakpoints possess microhomologies indicative of replication slippage or recombination based repair, whereas others possess no homology at the junctions. In the latter case, NHEJ must occur and likely involves structural features (e.g. attachment points on the nuclear matrix) that juxtapose free ends. Common fragile sites are A/T rich, possess various repeats and classes of repetitive DNAs (particularly LINE/LTR type elements), are gene-poor regions, and occupy large tracts of eukaryotic genomes, sometimes extending over hundreds of thousands of nucleotides [29], [30]. Our analyses of the SBC revealed that four of the five alleles harbor Athila/LTR retrotransposon elements at or near the breakpoints, which are A/T rich. The A/T rich sequences may contribute to secondary structure formation (see below) and could serve to anchor the chromatin fiber to MAR regions, which are known to be enriched for enzymes of DNA metabolism [31]. Indeed, the inhibition of topoisomerase I by camptothecin almost completely eliminates CFS breakage in cultured mammalian cells [32].

Fragile sites are found at the interface of chromosome R and G bands, classically defined as being early and late replicating, respectively [33]. Recent studies suggest that CFS activation (breakage) may be due to differential utilization of replication origins such that the CFS zone may experience replication stalling or fork collapse [34], [35], and secondary structures likely contribute to this process [36]. The paucity of replicon initiation in these regions necessitates the use of more distant origins and delays replicon completion, in part explaining their location at R/G boundaries. In Arabidopsis, high resolution profiling of replication in suspension cultured cells has revealed the locations of origins and their replication timing [37], [38]. The south breakpoint cluster in T12G13 is situated between two distantly separated origins that span 271 Kbp; for comparison, the interorigin median and mean distances along chromosome 4 are 51.1 and 77.2 kbp, respectively. We propose that the south breakpoint cluster possesses the hallmark features of metazoan chromosome fragile sites, harboring sequences that promote replication fork collapse, and that loss of DNA may be limited by association of local sequences with the nuclear matrix, where topoisomerase/ligase activities can coordinate repair by a NHEJ (or other) mechanism. In addition to the deletions associated with *BP/HY4*, this stochastic process may occasionally generate rearrangements and chromosomal fusion events that underpin chromosome evolution.

### Genome Variation among Accessions and Chromosome Evolution

Arabidopsis has been extensively used for comparative evolutionary genomics studies in plants. The 1001 Genomes Project has generated a wealth of sequence information, and numerous SNPs and indels are known to exist in many ecotypes [39]–[41]. The vast majority of this work has employed high throughput sequencing technologies, generating short sequence reads that are mapped onto scaffolds of reference genomes. While this strategy is useful and saturation can be achieved, it has two major disadvantages. First, some syntenic relationships may be masked, as many inversions and translocations cannot be accurately mapped. This is also the case for sequences associated with repetitive DNAs. Second, some copy number variants and their locations may go undetected and unrecognized, depending on the extent of divergence amongst them and their flanking sequences. While paired end mapping can be used to discover and verify structural variation [42], only through the sequencing of contiguous long clones (e.g. BACs) can such sequence anomalies be accurately located and quantified. In Arabidopsis, several such studies have revealed striking departures from the Columbia reference genome. For example, comparative sequence analysis of a region around 100 map units on chromosome 1 in Columbia vs. L*er* revealed that the region in Columbia is approximately 135 kbp, while the comparable region in L*er* is only 71 kbp [43]. This appears to be due to several gross rearrangement events including two different ecotype specific duplications, a deletion and several ecotype specific transposition events that ultimately led to the shorter L*er* region having fewer R genes than its Columbia counterpart [43]. In a similar vein, Lai et al. [44] resequenced a 371 kbp region of chromosome 3 from Columbia and L*er*, mapping these reads onto both the Columbia reference genome as well as the more recent Wellcome Trust generated L*er* draft genome, and discovered 61 misassemblies and large structural variants not represented by the draft genome. Lastly, the *quartet* mutation has afforded the possibility of tetrad analysis in Arabidopsis, and Lu and coworkers [45] sequenced the F_1_ genomes of a Col/L*er* cross, reporting numerous known and heretofore unknown SNPs and indels. Analysis of the indels revealed that a deletion of 19.2 kbp encompasses the region of the *bp-11* north breakpoint. These high resolution sequencing projects reveal that the genomes of Arabidopsis accessions are highly polymorphic and subject to rapid change as a result of normal meiotic recombination events and other rearrangements that may be due to replication errors and mobile element activity.

While the power and economics of high throughput sequencing technologies cannot be disputed, discovering and mapping large structural variation in comparative genomics studies ultimately will depend upon more painstaking mapping and the generation and analysis of long clones. Fluorescent in situ hybridization or chromosome painting offers a middle ground for analyzing gross chromosomal architecture. Our experiments reveal that the two most commonly used Arabidopsis ecotypes, Columbia and Landsberg *erecta*, possess at least two major structural differences on chromosome 4. The heterochromatic knob, which can be readily detected by DAPI staining, arose in Columbia via a pericentric inversion, moving a heterochromatic region towards the north end of the chromosome [13], [14]. South of the centromere, there appears to be an indel that generates a significant length difference between the two GREEN probeset signals located at 4.3 Mbp and 6 Mbp of the Columbia reference genome. The precise nature of this polymorphism is unknown, but a comparison of homologous regions in *A. lyrata* and *A. thaliana* (Columbia) indicates that on two contigs that span the region, *A. lyrata* possesses over 600 kbp more DNA than Columbia. It is possible that this region has persisted in the L*er* genome, accounting for the longer GREEN1/GREEN2 intervals that we observed. Given that similar polymorphisms are likely to exist on the other chromosomes, which could involve the mobilization of large blocks of sequence to perhaps distant sites (e.g. an inversion involving over 1 Mbp), map-based cloning of some genes could be complicated and require protracted efforts.

### Does the Chromatin State Determine the Integrity of the Genome?

Advances in chromatin immunoprecipitation have permitted high throughput approaches for elucidation of the histone codes associated with genome elements. Roudier et al., [19] conducted integrative epigenomic mapping in Arabidopsis, tracking 12 chromatin modifications to define four primary chromatin states associated with different coding and noncoding sequences. As in other organisms, the Arabidopsis pericentromeric DNA is enriched for several histone modifications, in particular methylation at H3K27, H3K9, and at H4K20 residues. In the context of maintaining genome integrity, H4K20 modifications are intriguing. In mammals, the H4K20 methylation state plays important roles in DNA damage repair as well as in class switch recombination (CSR) during the maturation of antibody producing cells [46]. CSR employs a NHEJ mechanism to exchange constant regions of immunoglobulin genes during B cell differentiation [47]. In mouse cells in which H4K20 methyl transferases are silenced, H4K20me1 accumulates, and this is associated with translocations and deletions of the IgH locus [46]. To our knowledge, there are no reports in Arabidopsis on the distribution and role of either di- or trimethylated H4K20, but these modifications might prove to associate with the instability that we observe.

## Methods

### Biological Materials

The alleles *bp-1*, *bp-2*, *bp-3*, and *bp-5* were described by Douglas et al. [8]. *bp-11* was obtained through ABRC (CS3161). Table S1 contains information on all characterized *bp* mutants. Plants were grown in environmental growth chambers with a 16 hr day/8 hr night cycle at 22°C under fluorescent lighting of approximately 100 µE/m^2^. Bacterial artificial chromosome clones were obtained from ABRC and DNA was prepared by employing Qiagen midi-prep columns.

### Molecular Techniques

General molecular techniques were carried out as described by Sambrook et al. [48]. Genomic DNA for PCR was prepared using Sigma GeneElute columns. A list of primers used for determining the presence or absence of a locus, as well as for use in inverse PCR and phase 5 PCR is given in Tables S2, S3, S4. Breakpoint junctions were cloned by employing inverse PCR. Genomic DNA was digested with a restriction enzyme that was shown by DNA gel blotting to give rise to an RFLP. The digested DNA was purified, diluted to approximately 1 µg/ml and subjected to overnight ligation to promote recircularization. Inverse PCR primers were then employed in PCR reactions to generate products containing the breakpoint junctions. These molecules were cloned into pJet1.2 (Fermentas) and sequenced (The Centre for Applied Genomics, Toronto).

For Southwestern analysis, a cDNA encoding the MAR binding protein AHL1 [15] was cloned by conducting RT-PCR on silique RNA, using oligo dT to prime first strand synthesis, and two primers: MAR Forward Nhe: 5′ AGGCTAGCGTCTTAAATATGGAGTCTACC 3′ and MAR Backward Bgl II: 5′ AAAGATCTGATTTCAAGTTACATTGACATTAATATCGG 3′. The underlined sequences represent engineered Nhe I and Bgl II sites that were used to clone the cDNA into the expression vector pRSET B (Invitrogen). Authenticated clones were mobilized into BL21 cells and expression of AHL1 induced by the addition of IPTG to a final concentration of 1 mM. After several hours of growth, induced and uninduced cultures were harvested by centrifugation and resuspended in Laemmli buffer (100 µl per 1 ml of culture), boiled, and stored at −20°C until needed. For Southwestern blotting, aliquots of protein extracts from induced and uninduced cultures were subjected to SDS-PAGE and transferred to nitrocellulose. The membrane was placed in 3% gelatin/TBS and kept at 4°C overnight. Protein refolding and blocking was carried out for 2 hours in a solution of 5% non-fat dry milk (Carnation), 20 mM Tris, pH7.6, 150 mM NaCl, 10 mM MgCl_2_, 0.25 mM DTT, 0.05% Tween-20, and 10 µg/ml salmon sperm DNA. DNA fragments to be used for probes were generated by PCR (see Table S6 for primer set information) and cloned into pJet1.2 (Fermentas). Probe DNA was prepared by fill-in reactions using alpha ^32^P-dCTP and the Klenow fragment of DNA polymerase I (Invitogen) on restriction fragments. Binding was performed in the refolding buffer except that the non-fat milk concentration was reduced to 0.5%. The membranes were gently agitated at room temperature for 2 hours in approximately 3 ml of liquid containing the radiolabeled probes, then washed four times in 20 mMTris pH7.5, 200 mM NaCl, 10 mM MgCl_2_, 0.05% Tween-20, 0.1% Triton ×100, 0.25 mM DTT and 10 µg/ml salmon sperm DNA. Autoradiography was then employed to detect binding. In no case was binding detected using uninduced extracts. Probe labeling was assessed by gel electrophoresis and autoradiography and all probes were deemed to be of comparable specific activity.

### Bioinformatics and Data Mining

The BLASTn search tool, the MEME algorithm (National Biomedical Computation Resource website; version 4.9.0; http://meme.sdsc.edu/meme/cgi-bin/meme.cgi; Accessed 2012 Nov 7 [49]) and the RepeatMasker algorithm (Institute for Systems Biology website, open-3.3.0. Available: http://www.repeatmasker.org/cgi-bin/WEBRepeatMasker. Accessed 2012 Nov 7. [50]) were employed to identify common motifs and repetitive elements. Potential secondary structures were determined by employing the Mold algorithm [51]. The EPIGARA database (Arabidopsis epigenetics and epigenomics group website; version 1.69; http://epigara.biologie.ens.fr/index.html. Accessed 2012 Nov 7.) of chromatin modifications was interrogated for locations of modifications and their proximity to the *bp* breakpoint junctions. Data was extracted from ChIP/chip studies conducted by Roudier et al. [19] and array based profiling of methylated DNA [52]. The pie charts in Figure 6 were generated by taking the IP/input log ratio for each region and dividing this by the sum of all IP/inputs for the region.

### Fluorescence In Situ Hybridization (FISH)

Staged floral buds of wildtype Columbia and the Columbia based *bp-5* and *bp-11* alleles, along with Landsberg *erecta* (L*er*) and the L*er* derived *bp-1* and *bp-2* alleles were used as the starting materials. Pachytene chromosome spreads were prepared and identified according to the method of Stronghill and Hasenkampf [53]. Spreads were then subjected to fluorescence in situ hybridization as described by Lysak *et al*. [54]. Five chromosome 4 probesets, comprised of bacterial artificial chromosomes (BACs), were used to determine the contour distances between these five sequences, which span chromosome 4 from 0.8 Mbp to 6.4 Mbp (AGI coordinates). Probes were generated by employing a Nick Translation Kit (Roche). Two of the five probesets (north of the centromere) were biotin-labeled BAC clones bracketing the heterochromatic knob region in Columbia: Red 1: BACs T7B11, T2H3 and Red 2: BACs T4B21, T1J1, T32N4. Detection of these signals was facilitated by goat anti-biotin antibodies (Vector Laboratories) and a secondary donkey anti-goat Cy3 conjugated antibody (Jackson Immunoresearch). Three additional probesets were DIG-labeled BAC clones (south of centromere), bracketing the region of the *BP* locus: Green 1: BACs F28D6, T3E15; Green 2: BACs T15G18, T25P22 and Green 3: T9A4, F24G24. These hybridization signals were detected by employing a mouse anti-DIG primary antibody and a donkey anti-mouse FITC conjugated secondary antibody (Jackson Immunoresearch). Spreads were examined using a Zeiss axiophot epifluorescent microscope and a Plan-Neofluar 100×/1.3NA oil immersion objective lens. Northern Eclipse 5.0 software was used to capture images and measure the distance between FISH signals. Merged images were created using Photoshop CS5 software.

## Supporting Information

Figure S1 Motifs identified by MEME analysis. One kilobase of sequence north and south of the breakpoint for each *bp* allele (see Figure 3) was uploaded to the MEME server (National Biomedical Computation Resource website; version 4.9.0; http://meme.sdsc.edu/meme/cgi-bin/meme.cgi; Accessed 2012 Nov 7 [49]) and default search parameters were employed. The top part of each figure shows the consensus sequence logo with the height of the letters proportional to the consensus nucleotide at each position. The bottom part of each figure presents the individual occurrences of the motif. For each motif, the allele is identified by the ‘Name’ column and the location of the motif on the plus or minus strand is given, along with the starting nucleotide coordinate. The p-values for each sequence comparison represent the probability of a randomly generated sequence of the same length having a match score at least as large as the motif sequence.
(PDF)

Figure S2 Mfold diagrams of potential secondary structures. Predicted secondary structures within or near the *bp* breakpoints are shown as determined by Mfold analysis (The RNA Institute Website; mfold web server: 1995–2012; http://mfold.rna.albany.edu/?q=mfold/DNA-Folding-Form; Accessed 2012 Nov 7). The north donor sequences, except where noted in *bp-2* north, consist of 1000 nucleotides before the north breakpoint which is retained sequence, and is followed by 1000 nt of DNA which is deleted. Similarly, the south breakpoint sequences begin with 1000 bp of sequence that is deleted and following the junction, contain 1000 nt of sequence that is retained. Default search parameters were employed. For brevity, the only structures shown are those near the breakpoints. The red arrows point to the junction breakpoint in each sequence.
(PDF)

Figure S3 Detailed epigenomic map of the south breakpoint cluster. The AGI coordinates of the breakpoint junction regions were uploaded as added tracks to the Epigara database (Arabidopsis epigenetics and epigenomics group website; version 1.69; http://epigara.biologie.ens.fr/index.html. Accessed 2012 Nov 7.) Epigenetic data in the chr4 tiling array was then interrogated for the histone modifications shown. The location of the *bp* breakpoints is shown at the top and the vertical line allows the viewer to determine if enrichment exists along each modification line.
(PDF)

Figure S4 Chromosome map of Arabidopsis deletion mutants. The Web of Science database was queried with the search terms: Arabidopsis/mutant/deletion to discover literature reports on deletion mutants. In parallel, the TAIR germplasm search engine was used to identify deletion mutants. Literature reports and TAIR database entries were examined for congruence and duplicates were discarded. Deletions of over 25 bp were used as a cutoff threshold and the AGI numbers for each mutant were identified by employing the TAIR database. The list of 120 AGI numbers was uploaded to the TAIR chromosome map tool to display deletion locations. Where more than a single deletion mutant exists for a gene, the AGI number is replaced by the gene identifier, and the number in parentheses indicates the number of independent deletions for that locus (e.g. *bp* (5) represents the five deletion alleles we report here).
(PDF)

Table S1 Summary of characterized *bp* alleles.
(PDF)

Table S2 Primers used to localize deletion breakpoint regions.
(PDF)

Table S3 iPCR primer sets.
(PDF)

Table S4 Phase 5 primer sets.
(PDF)

Table S5 List of genes deleted in *bp* mutants.
(PDF)

Table S6 Primers used to generate southwestern blot probes.
(PDF)
