# Supplementary material for: Chromosome Fragile Sites in Arabidopsis Harbor Matrix Attachment Regions That May Be Associated with Ancestral Chromosome Rearrangement Events
Source: PLoS Genet. 2012 Dec 20;8(12):e1003136. doi: 10.1371/journal.pgen.1003136 (PMC3527283; doi:10.1371/journal.pgen.1003136)
Supplement: Figure S1 — Motifs identified by MEME analysis. One kilobase of sequence north and south of the breakpoint for each bp allele (see Figure 3) was uploaded to the MEME server (National Biomedical Computation Resource website; version 4.9.0; http://meme.sdsc.edu/meme/cgi-bin/meme.cgi; Accessed 2012 Nov 7 [49]) and default search parameters were employed. The top part of each figure shows the consensus sequence logo with the height of the letters proportional to the consensus nucleotide at each position. The bottom part of each figure presents the individual occurrences of the motif. For each motif, the allele is identified by the ‘Name’ column and the location of the motif on the plus or minus strand is given, along with the starting nucleotide coordinate. The p-values for each sequence comparison represent the probability of a randomly generated sequence of the same length having a match score at least as large as the motif sequence. (PDF) [file pgen.1003136.s001.pdf]

Figure S1: Motifs identified by Meme Analysis

Motif 1:

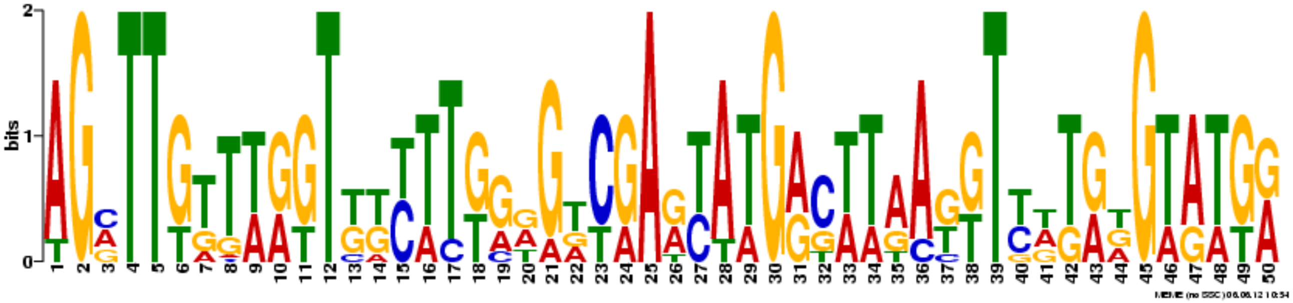

Name	Strand	Start	*p*-value	Sites		
bp2N	+	249	2.79e-22	AGCAAGAAGA	AGCTTGGTTAGTGTTTTGGAGTCGAATATGACTTGATGTCATGTGTATGA	TTGAGTATAA
bp2N	+	81	4.79e-21	AGCTAGAAGA	AGCTTGGNTAGTGTTTTGGAGTCGAATATGACTTGATGTCATGTGTATGA	TTGAGTATAA
bp11N	+	288	2.05e-19	AGATTAAGGC	AGGTTGTTAGGTTGCATTGTGACGAGTATGGCTAAAGTTTTTAAGTAAGG	GTCTATCATC
bp2N	+	426	2.27e-19	AGCAAGAAGA	AGCTTGATTAGTGTTTTGGAGTTGAATATGACTTTATGTCATGTGTATGA	TTGAGTATAA
bp5N	-	309	2.80e-19	AGATTAAGGC	AGGTTGTTAGGTTGCATTGTGACGAGTATGGCTAACGGTTTTAAGTAAGG	GTCTATCACT
bp11N	+	446	1.90e-17	CTTCAACTTA	AGATTTTTTGTTTGCTTGAGGGCAAGCAAGAGATAAGTTTGGGGGAGTTG	ATAACACAGT
bp5N	-	151	8.00e-16	TTTCAACTTA	AGATTTTTTGTTTACTCGAGGGCAATCAAGAGATAAGTTTGGGGGAGTTG	ATAACACTGT
bp5N	-	1012	2.54e-15	CATTTGTGTG	TGATTGTGAGGTCTTTTTCGATTGAGCTTGGTATAACGTGTTAGGTATGA	GCCGACAAGA

Motif 2:

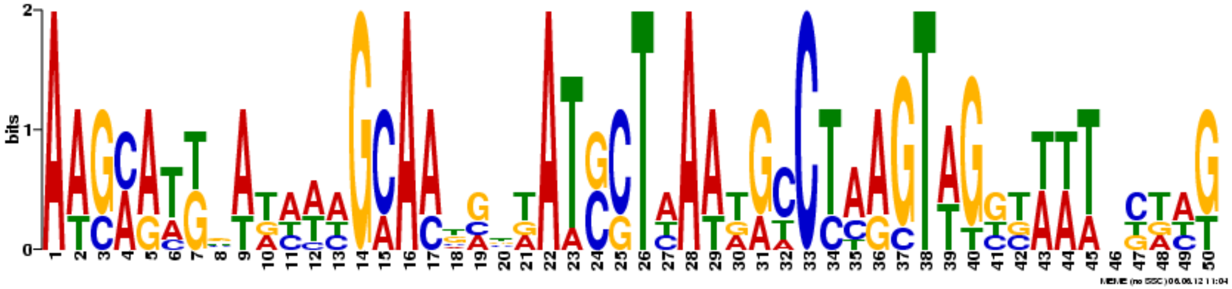

Name	Strand	Start	*p*-value	Sites
bp2N	+	484	2.34e-18	GATTGAGTAT AAGAATTTAAATCGCAATCTTATCCTAAAAGCCTAAGTAGTGTTTCCTTG TTAGAATATA
bp11N	-	355	5.51e-18	TCAAAAACCG AAGCATGCATATAGAACGGGTATGCTCAATGTCCTGGTAGCTAATTCTAG GTTGCAAGAT
bp5N	+	242	7.39e-17	TCAAAAATCG AAGCATGCATATAGAACAGGTAAGCTCAATGTCCAGGTAGCTAATTCTAG GTTGCAAAGT
bp2N	+	139	1.31e-16	GATTGAGTAT AAGAACTTAAACCGCAACCATATCCTAAAAGCCTAAGTAGTGTTTCCTTG TTAGAATATA
bp11N	-	180	2.67e-16	ATTAAGCACG AACAGTGGATCAAGCAATGCAATGGTTATGACCTCAGTTGGTTTTGGGCT TAGGCTAAGA
bp11N	-	589	1.05e-15	ACACCAAAAG ATGCAATATGTATGCAAGATGATCCTAAATGCCTAAGTTGGCAAAATAAG CTAAATGTGA
bp5N	+	9	1.42e-15	CACCAAAAA ATGCAATGTGTATGCAAGATGATCCTAAATGCCTAAGTATGCAAAATAAG CTAAAATGGT
bp5N	+	380	8.68e-15	TTTAAGCACG AACAGTGGATCAAGCAATGCAATGGTTATGAACTCACTTGGTTTTGGGCT TAGGCTAAGA

Motif 3:

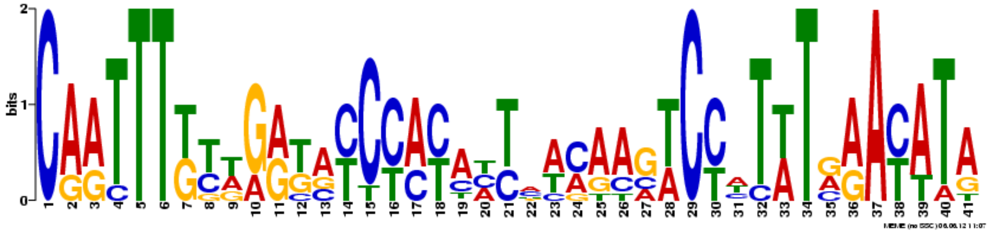

Name	Strand	Start	*p*-value		Sites	
bp1N	+	81	3.95e-16	AAATAACAAA	CAATTTTTTGGTGTCCATAATAACAAGTCCWTTTAAATATA	TCCATTCGGA
bp1N	+	280	6.85e-16	AATCATCAAA	CAATTTTTTGGCATCCATAATAACAAGTCCATTTAAATATA	TCCATTCAGA
bp1N	-	223	1.69e-15	GATTTTCTTT	CAGCTTGCAGAGACCTCCACCXTCAAGACCTTATGGACATG	GAACTCTGGT
bp1N	+	481	2.74e-15	AATCAACAAA	CAATTTGTTGGTGTCCATAATAACGAATCCATTTAAATATA	TCAATTCGGA
bp1N	-	422	4.82e-14	TTTTTTTTTT	CAATTTGCAGAGACTTCCACCCTCAAAACCXTATGGATATG	GAACTTTGGT
bp1N	-	23	1.20e-13	TTTTTTTTTA	CAGTTTGCAGAGACCTCCXCCTCGGAGACTTTATGGACATA	GAAATTATAC
bp5N	-	201	1.30e-13	ATGCATGCTT	CGATTTTTGAATCCCCACCTTCAAACCTCTCTTTCAACTTA	AGATTTTTTG
bp11N	+	405	4.66e-13	ATGCATGCTT	CGGTTTTTGAATCCCCACCTTCAAACCTCTCCTTCAACTTA	AGATTTTTTG
bp1S	-	233	4.11e-12	CTTCTATTTC	CAATTTTGTGGTATCCATTTTGTATTGTCCATATGAACAAT	GTACTTTTGC

Motif 4:

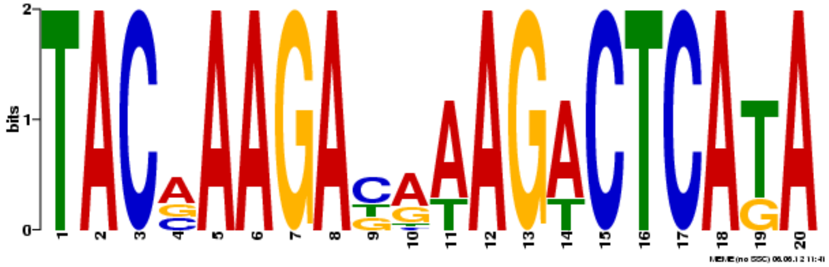

Name	Strand	Start	*p*-value	Sites
bp2N	+	542	1.10e-11	TGTTAGAATA TACGAAGACAAAGACTCATA TGGACTTCGG
bp2N	+	18	3.42e-11	GTTAGAAGGC TACAAAGACNAAGACTCATA TGGACCNTTG
bp2N	+	364	7.62e-11	CGTTAGAAGA TACAAAGATAAAGACTCATA TGGACTTCGG
bp2S	+	1076	3.89e-10	GAGGGAATCA TACCAAGAGGTAGTCTCAGA CCAGCATTAT

Motif 5:

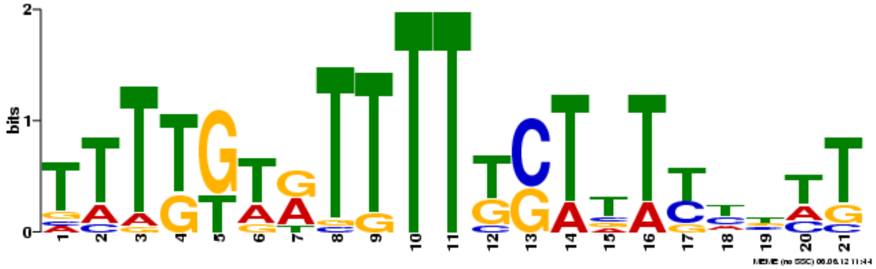

Name	Strand	Start	*p*-value	Sites		
bp1S	-	71	1.44e-08	AATTTAATAA	TTTTGGGTTTTGCTTATCCTT	ATAAATCATA
bp1S	-	187	3.26e-08	TGGTGACCAT	TTTTGTATGTTGGTTTCCTCT	TTTGTACCCA
bp1N	-	877	1.03e-07	ATTTTGATGG	TATGGAATTTTTCTTTCACTT	AGGGGATTGG
bp1S	+	1294	1.53e-07	AATCAAAATA	TTTTGTATTTTTCAATTTGTT	AGAGATAAAA
bp11S	+	1666	1.74e-07	TTGTGCTCTG	TTTTGTGGTTTTGTTTCATTT	TTAGCTTAAT
bp3N	-	609	3.97e-07	TTCCATCTTT	GCTTGTGTTTTGCTTTTCTCC	AGCCTAGATA
bp3N	-	249	3.97e-07	ACCTTGTCGC	TTTGTGGTTTTTCATTCACTT	GGGCCTTAAA
bp5N	-	1712	8.31e-07	ATACCATTGG	CTTTTTATTTTTCTTTGTGTG	TTTTGTGTTT
bp3S	+	87	8.31e-07	CAATAGAAAA	TTTTGAGTTTTGGATACTCCG	GGTTTACCCG
bp5N	+	1558	1.01e-06	TCAAATTTTT	TATGGTTTTTTTCTATGCCTT	AGATTGATGC
bp5N	-	1691	1.11e-06	TCTTTGTGTG	TTTTGTGTTTTGGTCTTGAAT	TTTTCCTCAG
bp11S	-	976	1.23e-06	TGATATATGA	TATTGTATTTTCCTGTTTGAG	TATATTTTCC
bp11S	+	1168	2.10e-06	ATATGATTAT	TTTGTTGTTTTTGTGATATAT	ATTGTATTTA
bp5S	+	1345	2.10e-06	TTCTCTTCTT	TTATGTACTTTGGTTTTTGTT	ATTCGAAAGA
bp5S	-	1196	2.28e-06	ATATTAAGAA	GTTGGGGTTTTCCTCATTGAT	CACCAAAATG
bp3S	+	1255	2.28e-06	AAATTTCAAT	TTGGTAATTTTTGTTTTTGTT	TGAATTTACA
bp11N	+	1085	2.70e-06	TAGCTTTTAT	CCTTTAGTTTTTCTTTGTGTT	CTTGAATCTT
bp1S	+	1498	3.17e-06	TCCATCCAAA	TATTTTGTTTTTGTCATATCT	TAATATGTAA
bp11S	+	1746	3.43e-06	TGCATAGAAG	GTATGAATTTTTCTTTTCTAG	ATATATAAGG
bp2S	-	365	3.71e-06	TCCGTCGTGG	ATTGGTATGTTTCAGTCCTCT	CTACTATATA
bp1S	-	1351	3.71e-06	TGTTAAAGTA	TTTTGTATTTTTCAATTAATG	GCATTCGATT
bp5S	-	1239	4.99e-06	ATTCTAAGGG	TATGTTTTGTTGGTTTCCTAT	TTATATCTTT
bp1N	-	638	4.99e-06	TGGCGGCTTG	ATTTGATTTTTGCTCTCTCTC	ATGTGTCAAA
