# Supplementary material for: Chromosome Fragile Sites in Arabidopsis Harbor Matrix Attachment Regions That May Be Associated with Ancestral Chromosome Rearrangement Events
Source: PLoS Genet. 2012 Dec 20;8(12):e1003136. doi: 10.1371/journal.pgen.1003136 (PMC3527283; doi:10.1371/journal.pgen.1003136)

Figure S2: Mfold diagrams of potential secondary structures. Predicted secondary structures within or near the *bp* breakpoints are shown as determined by Mfold analysis (http://mfold.rna.albany.edu/?q=mfold/DNA-Folding-Form). The north donor sequences , except where noted in *bp-2* north, consist of 1000 nucleotides before the north breakpoint which is retained sequence, and is followed by 1000nt of DNA which is deleted. Similarly, the south breakpoint sequences begin with 1000bp of sequence that is deleted and following the junction, contain 1000nt of sequence that is retained. Default search parameters were employed. For brevity, the only structures shown are those near the breakpoints. The red arrows point to the junction breakpoint in each sequence.

bp-1 north: no significant secondary structures identified.

bp-2 north: nucleotide 602 is the north breakpoint.

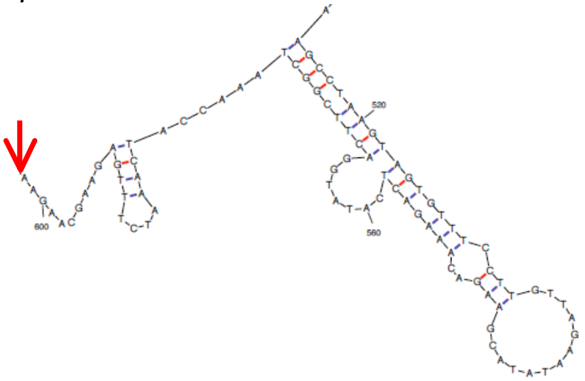

bp-3 north

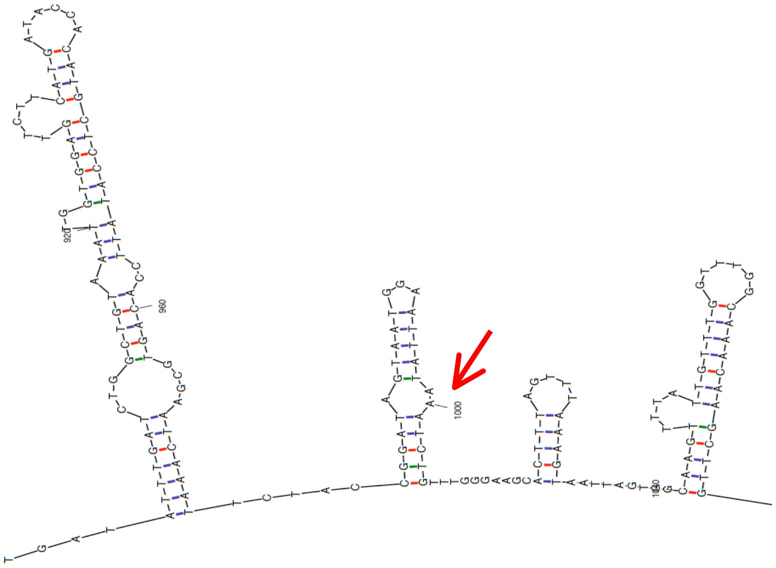

bp-5 north:

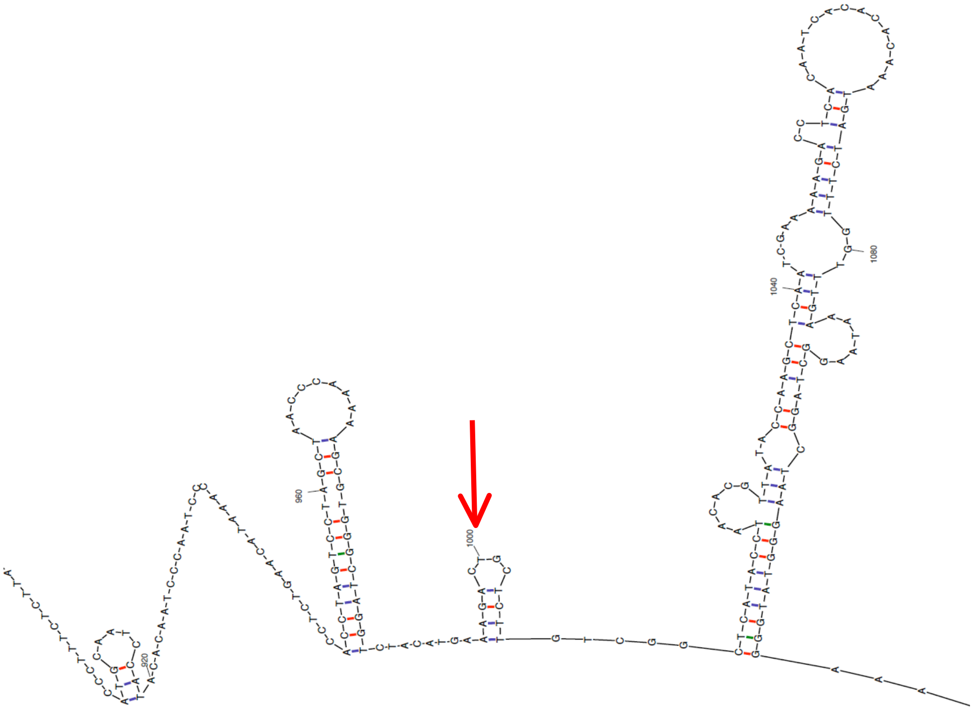

bp-11 north

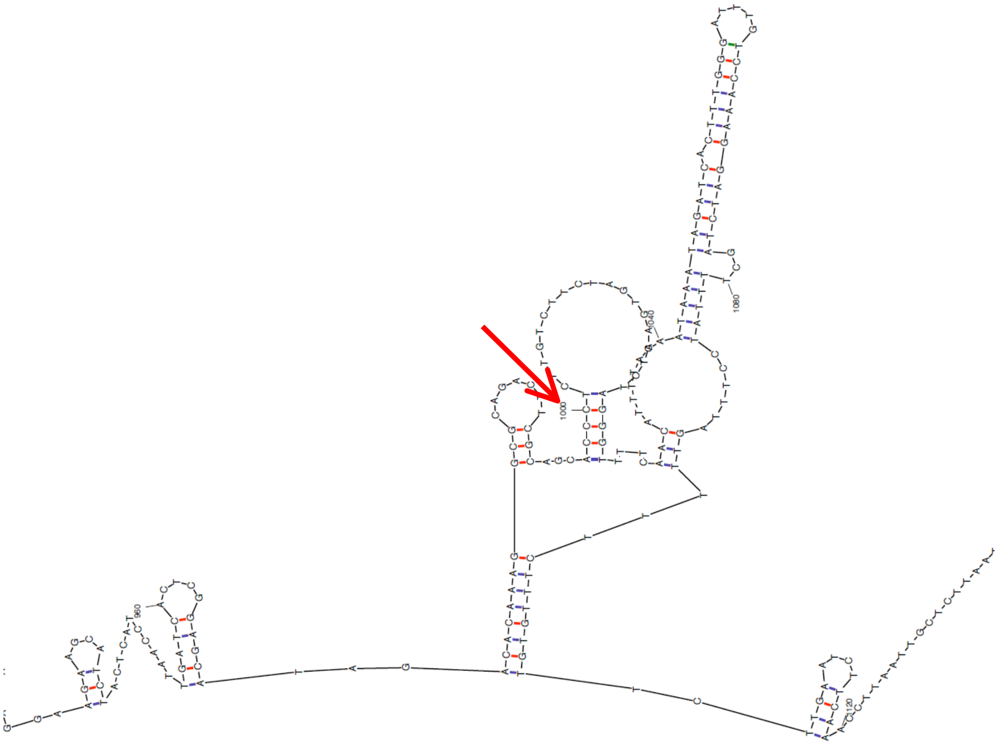

bp-1 south

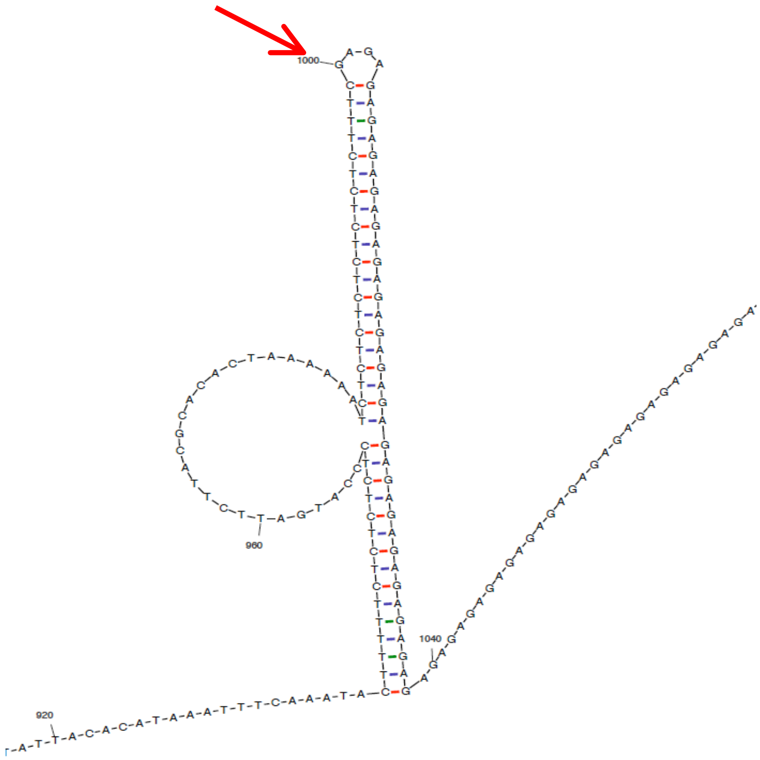

bp-2 south

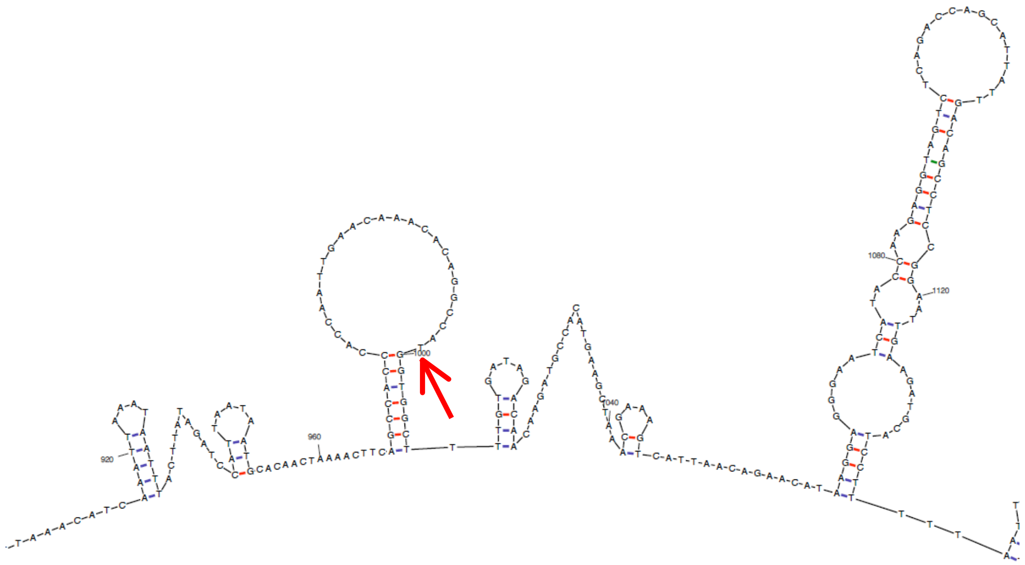

bp-3 south

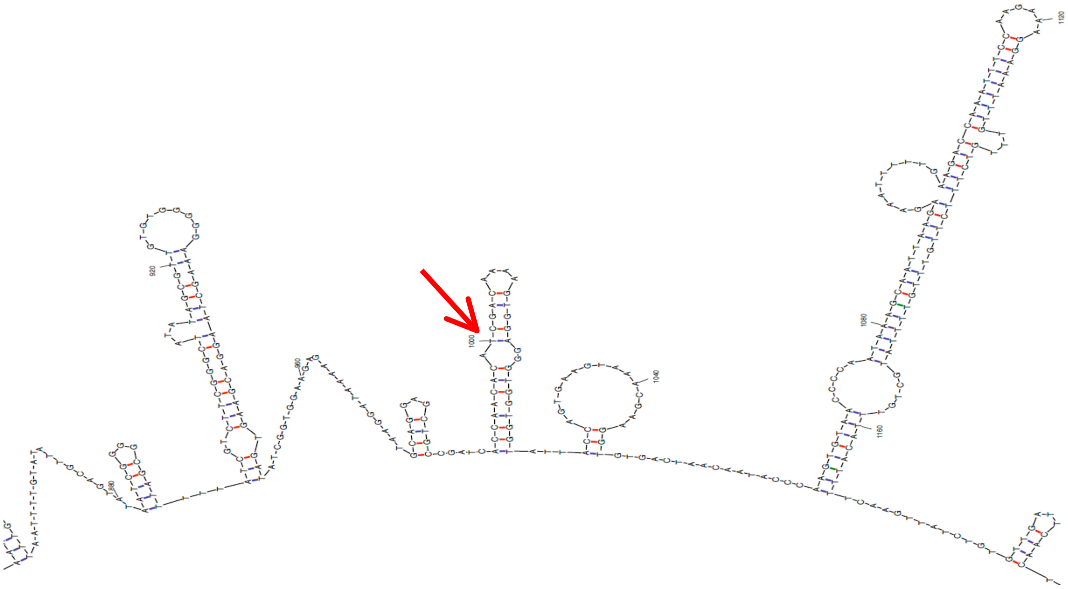

bp-5 south

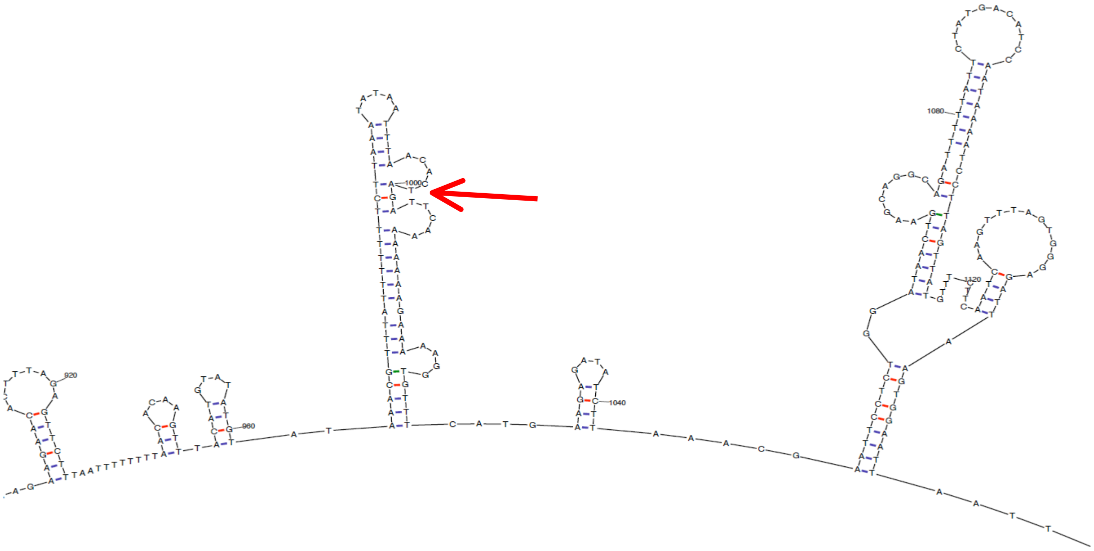

bp-11 south

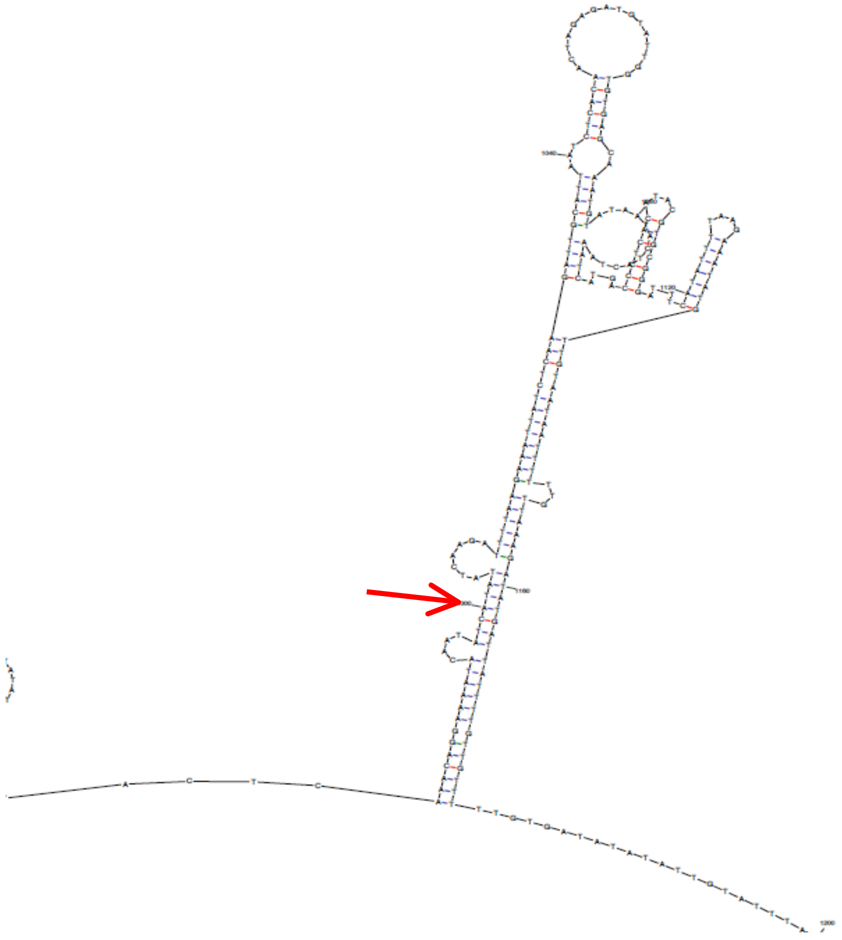

Supplement: Figure S2 — Mfold diagrams of potential secondary structures. Predicted secondary structures within or near the bp breakpoints are shown as determined by Mfold analysis (The RNA Institute Website; mfold web server: 1995–2012; http://mfold.rna.albany.edu/?q=mfold/DNA-Folding-Form; Accessed 2012 Nov 7). The north donor sequences, except where noted in bp-2 north, consist of 1000 nucleotides before the north breakpoint which is retained sequence, and is followed by 1000 nt of DNA which is deleted. Similarly, the south breakpoint sequences begin with 1000 bp of sequence that is deleted and following the junction, contain 1000 nt of sequence that is retained. Default search parameters were employed. For brevity, the only structures shown are those near the breakpoints. The red arrows point to the junction breakpoint in each sequence. (PDF) [file pgen.1003136.s002.pdf]
