# Supplementary material for: Chromosome Fragile Sites in Arabidopsis Harbor Matrix Attachment Regions That May Be Associated with Ancestral Chromosome Rearrangement Events
Source: PLoS Genet. 2012 Dec 20;8(12):e1003136. doi: 10.1371/journal.pgen.1003136 (PMC3527283; doi:10.1371/journal.pgen.1003136)

Figure S3: *bp5*N, *bp2*S, *bp1*S, *bp11*S, *bp5*S and *bp3*S (part1)

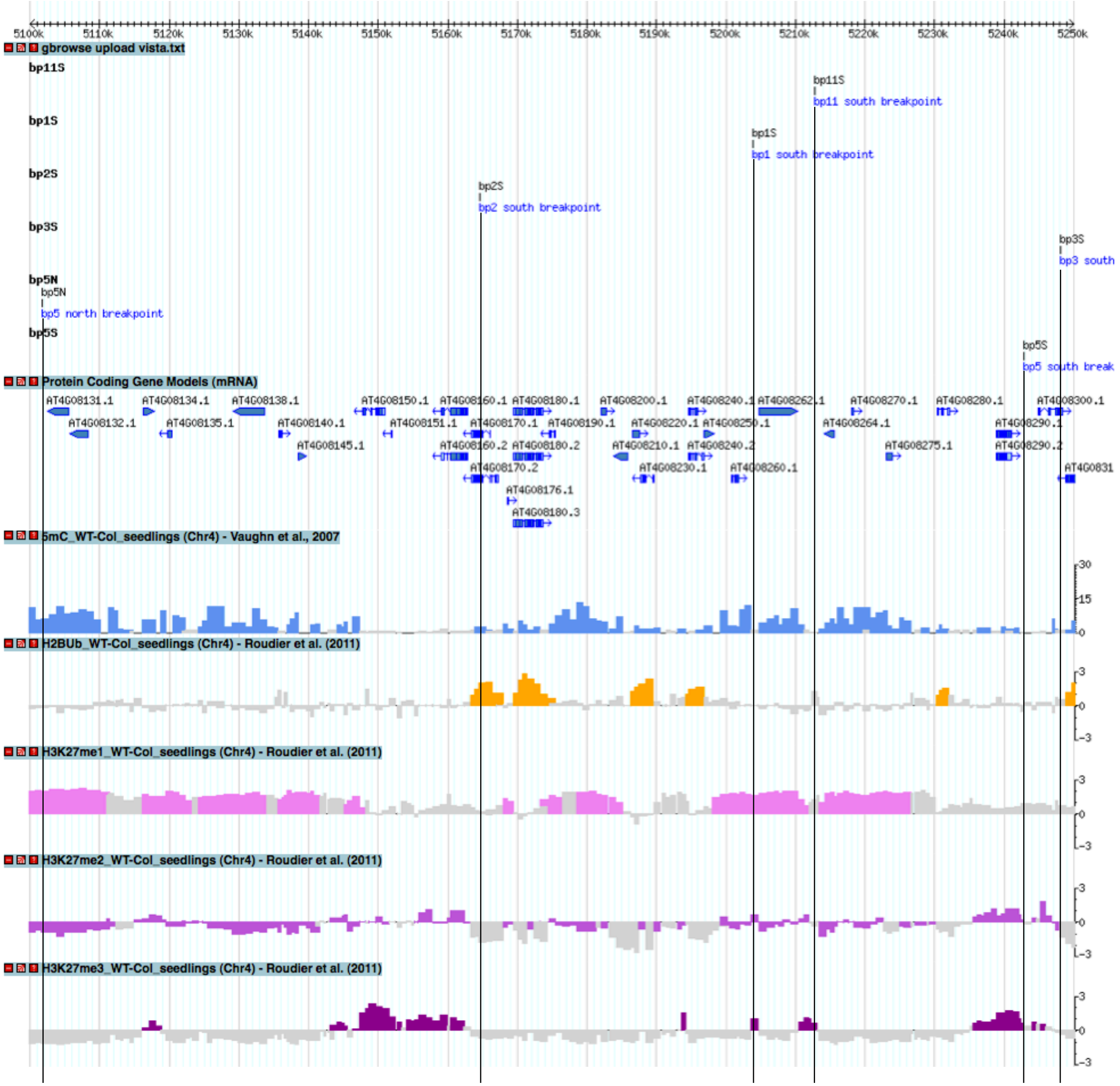

*bp5*N, *bp2*S, *bp1*S, *bp11*S, *bp5*S and *bp3*S (part 2)

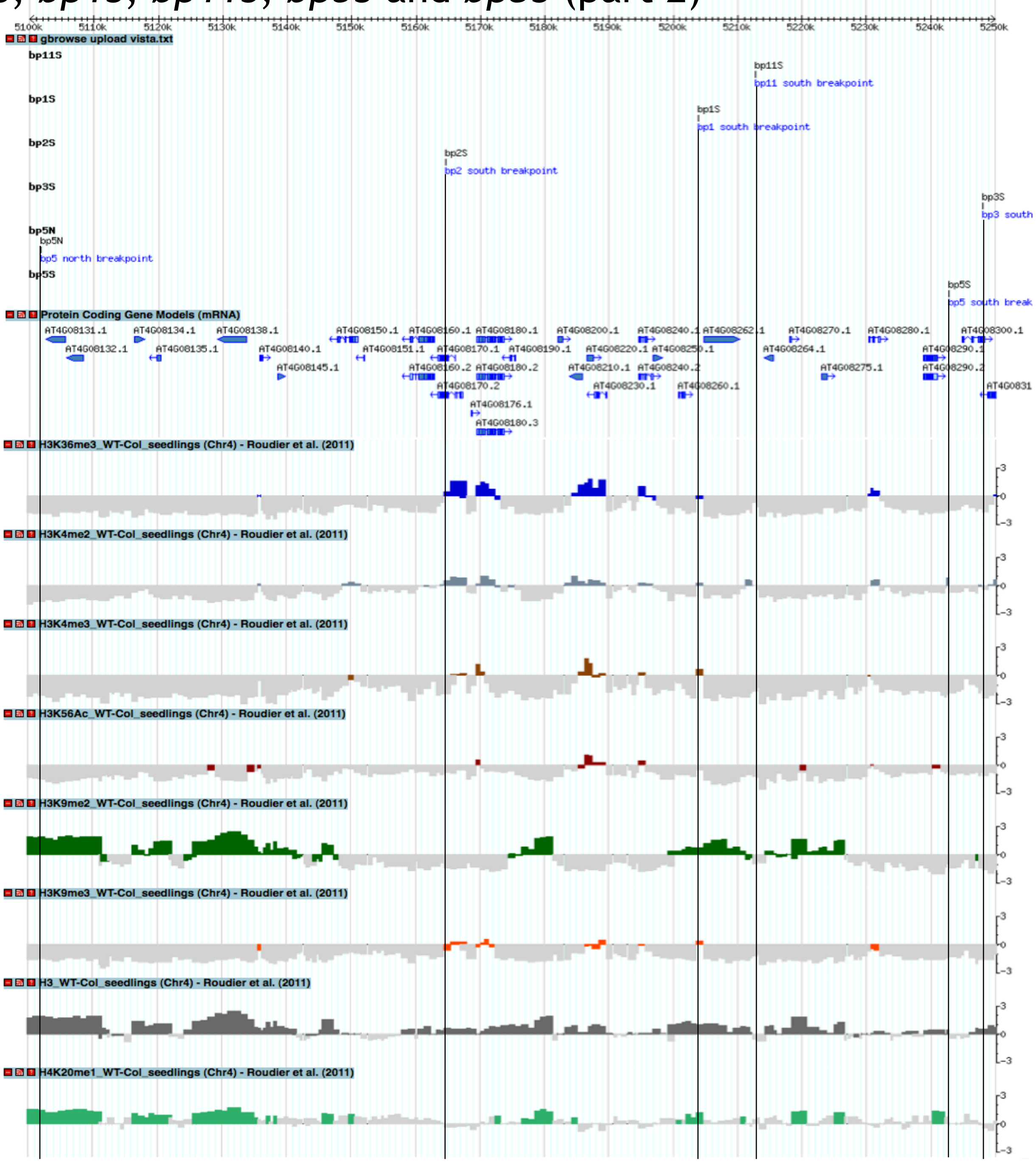

bp11 north (part 1)

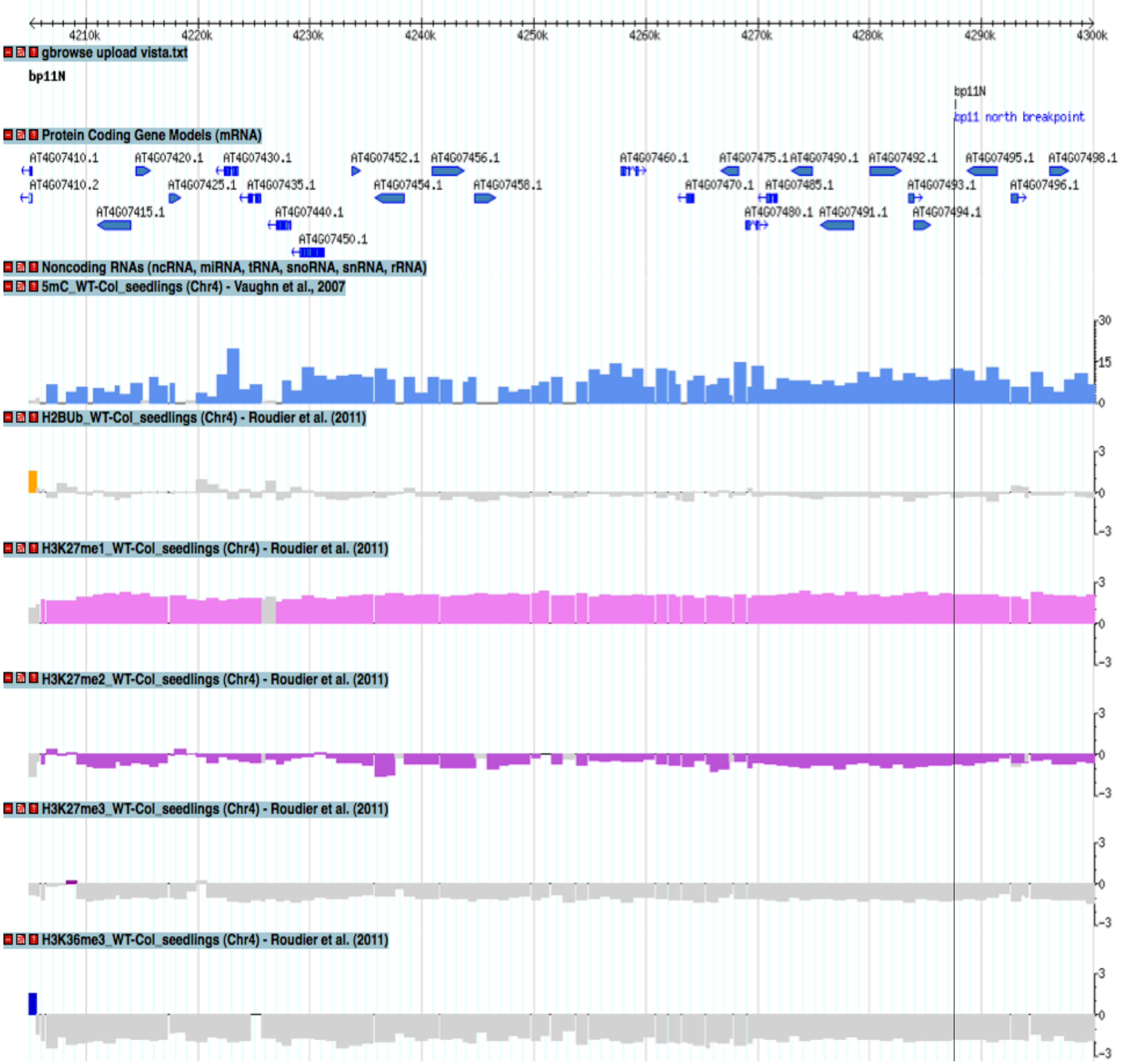

bp11 north (part 2)

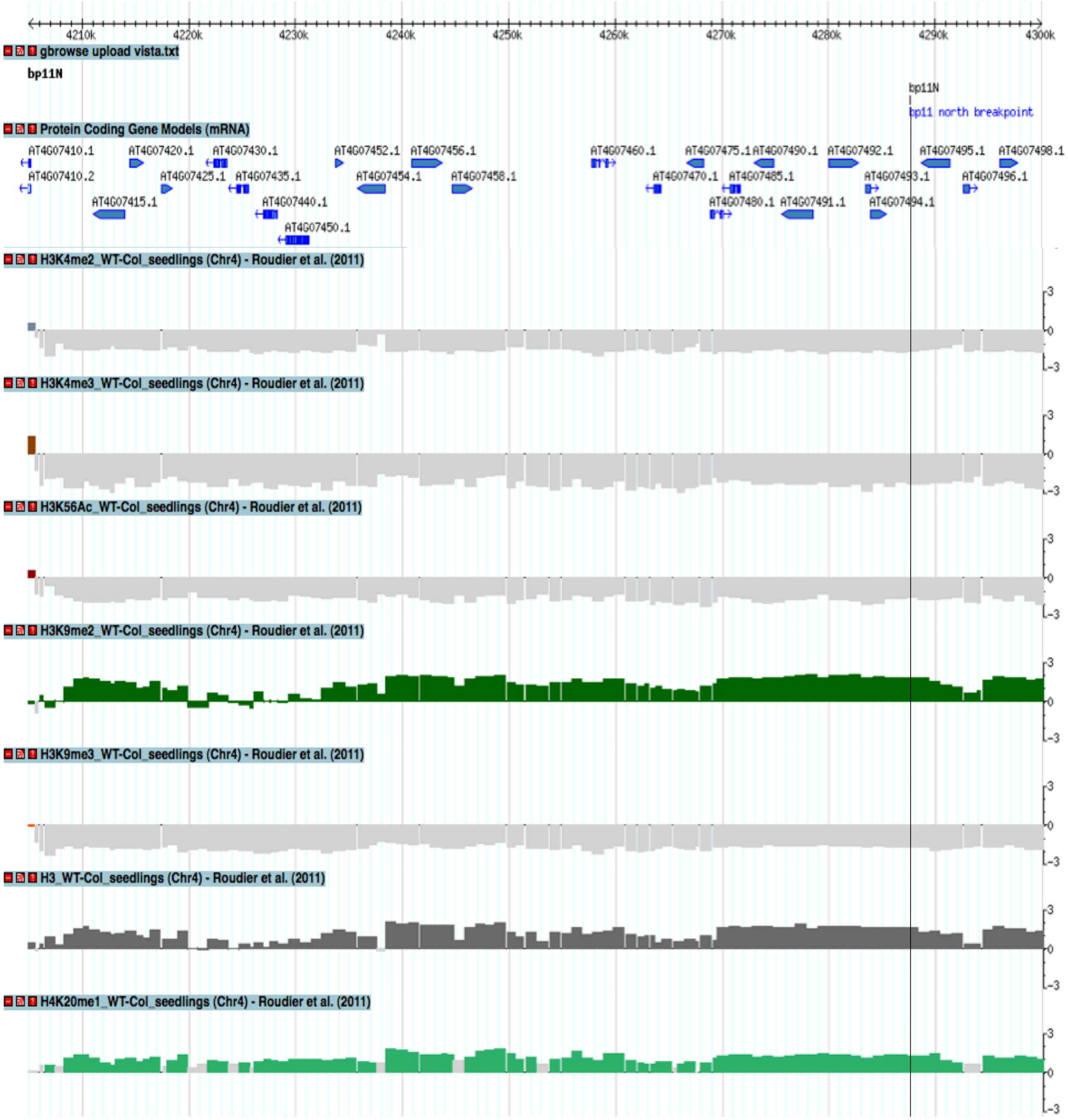

bp3 north (part 1)

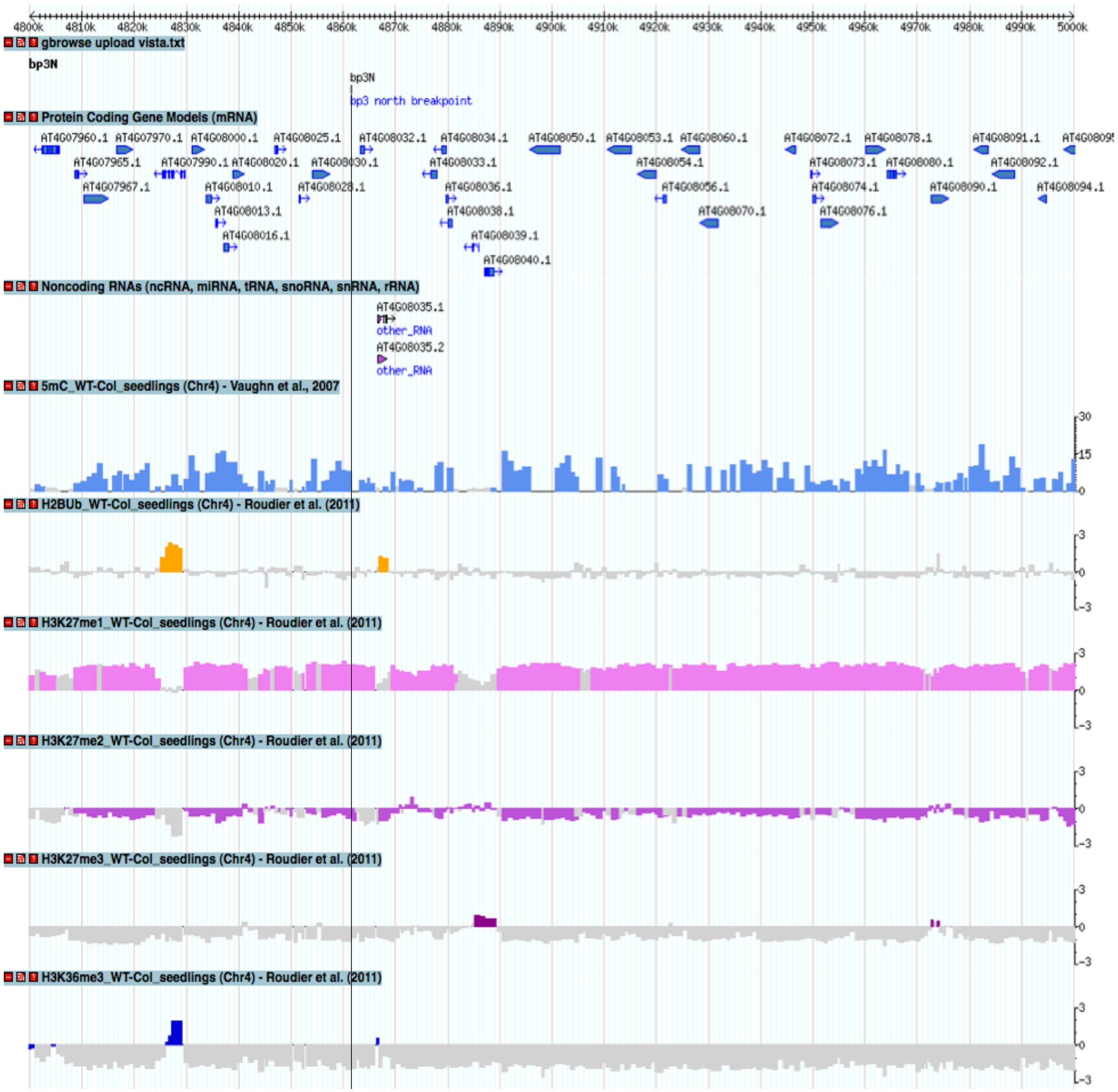

bp3 north (part 2)

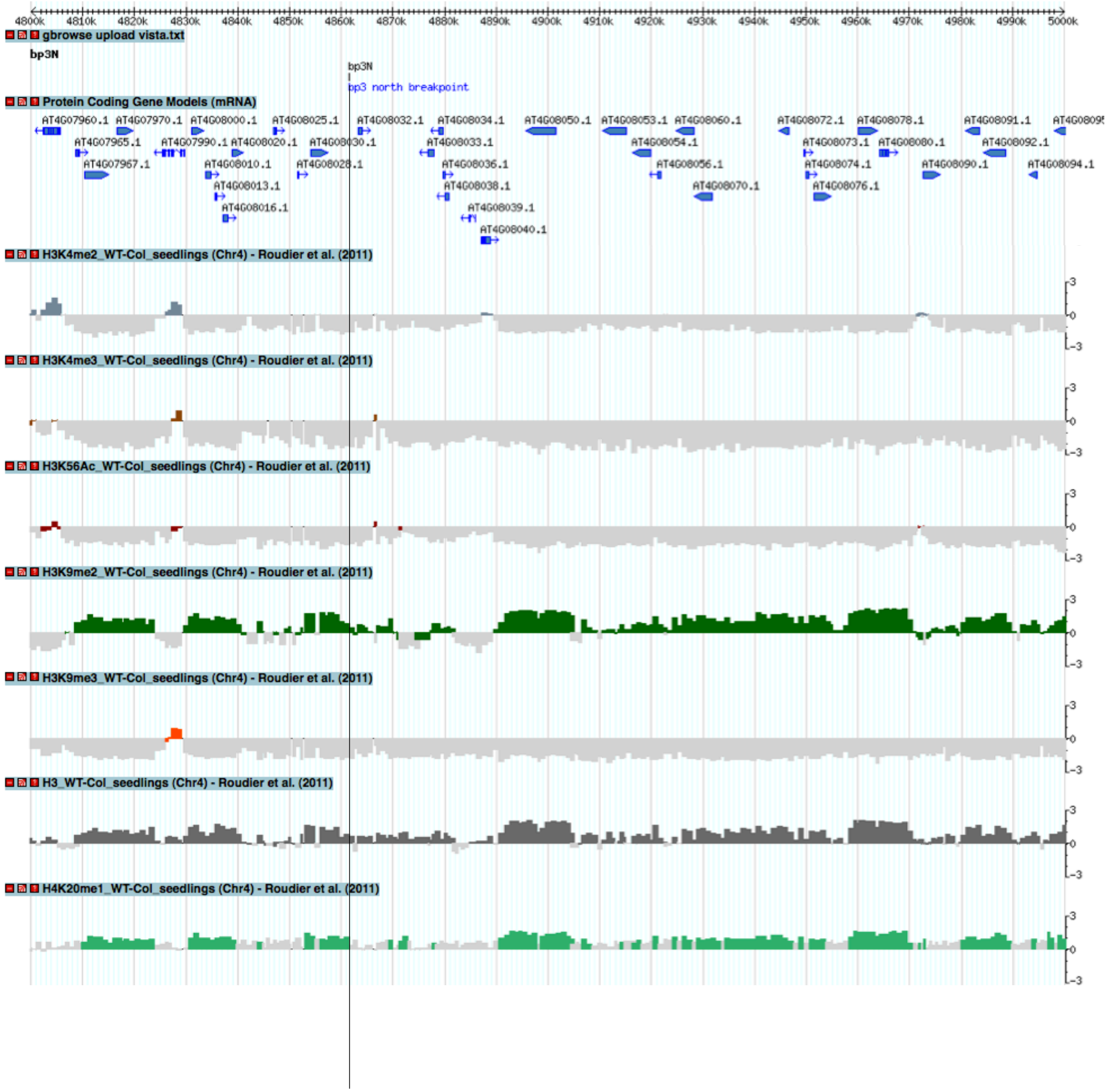

Supplement: Figure S3 — Detailed epigenomic map of the south breakpoint cluster. The AGI coordinates of the breakpoint junction regions were uploaded as added tracks to the Epigara database (Arabidopsis epigenetics and epigenomics group website; version 1.69; http://epigara.biologie.ens.fr/index.html. Accessed 2012 Nov 7.) Epigenetic data in the chr4 tiling array was then interrogated for the histone modifications shown. The location of the bp breakpoints is shown at the top and the vertical line allows the viewer to determine if enrichment exists along each modification line. (PDF) [file pgen.1003136.s003.pdf]
