# Supplementary figures and images for: Chromosome Fragile Sites in Arabidopsis Harbor Matrix Attachment Regions That May Be Associated with Ancestral Chromosome Rearrangement Events
Source: PLoS Genet. 2012 Dec 20;8(12):e1003136. doi: 10.1371/journal.pgen.1003136 (PMC3527283; doi:10.1371/journal.pgen.1003136)

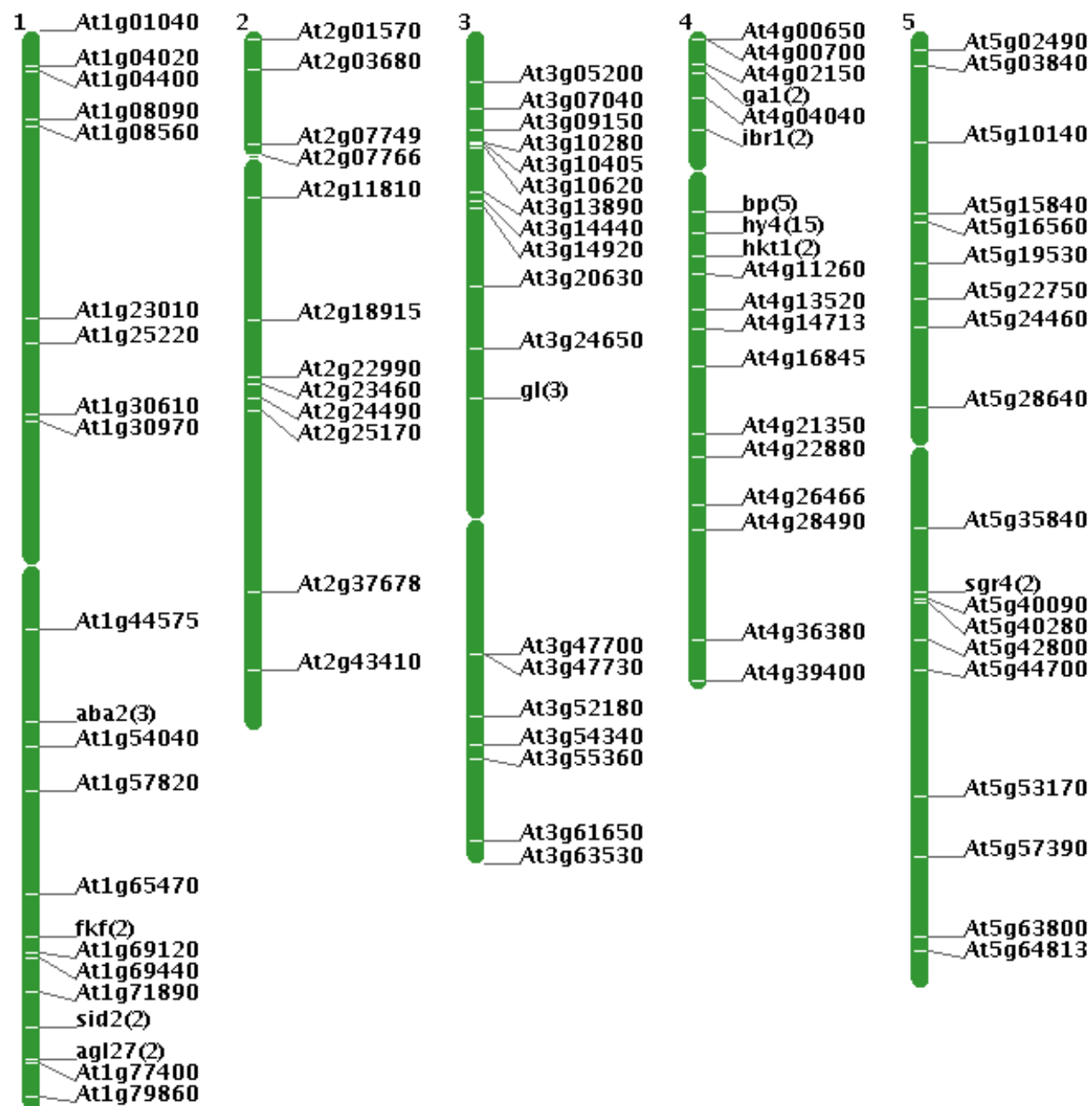

Supplement: Figure S4 — Chromosome map of Arabidopsis deletion mutants. The Web of Science database was queried with the search terms: Arabidopsis/mutant/deletion to discover literature reports on deletion mutants. In parallel, the TAIR germplasm search engine was used to identify deletion mutants. Literature reports and TAIR database entries were examined for congruence and duplicates were discarded. Deletions of over 25 bp were used as a cutoff threshold and the AGI numbers for each mutant were identified by employing the TAIR database. The list of 120 AGI numbers was uploaded to the TAIR chromosome map tool to display deletion locations. Where more than a single deletion mutant exists for a gene, the AGI number is replaced by the gene identifier, and the number in parentheses indicates the number of independent deletions for that locus (e.g. bp (5) represents the five deletion alleles we report here). (PDF) [file pgen.1003136.s004.pdf]
