# Supplementary material for: Chromosome Fragile Sites in Arabidopsis Harbor Matrix Attachment Regions That May Be Associated with Ancestral Chromosome Rearrangement Events
Source: PLoS Genet. 2012 Dec 20;8(12):e1003136. doi: 10.1371/journal.pgen.1003136 (PMC3527283; doi:10.1371/journal.pgen.1003136)
Supplement: Table S1 — Summary of characterized bp alleles. (PDF) [file pgen.1003136.s005.pdf]

Table S1: Summary of characterized *bp* alleles

Allele	Background	Mutagen	Mutation	Reference
bp-1	Ler	EMS	Deletion	Koornneef et al., 1983
bp-2	Ler	FN	Deletion	Douglas et al., 2002
bp-2#	RLD/Ler	T-DNA	Point mutant	Venglat et al., 2002
bp-3	Ler	FN	Deletion	Douglas et al., 2002
bp-4	Ler	EMS	Point mutant	Douglas et al., 2002
bp-5	Col	T-DNA	Deletion	Douglas et al., 2002
bp-6	Ler	EMS	Point mutant	Byrne et al., 2002
bp-7	Ler	EMS	Point mutant	Byrne et al., 2002
bp-8	Ler	EMS	Point mutant	Byrne et al., 2002
bp-9	Col	transposon	Insertion	Kanrar et al., 2006
bp-10	Col	EMS	Point mutant	Ikezak et al., 2010
bp-11	Col	X-rays	Deletion	Redei, unpublished; this work

#the *bp-2* mutant reported by Venglat was isolated concomitantly with the *bp-2* mutant we discovered, hence there are two known *bp-2* mutants.

References:

Byrne ME, Simorowski J, Martienssen RA (2002) ASYMMETRIC LEAVES1 reveals knox gene redundancy. Development 129, 1957-1965.

Douglas SJ, Chuck G, Dengler RE, Pelecanda L, Riggs CD (2002) KNAT1 and ERECTA regulate inflorescence architecture in Arabidopsis. Plant Cell 14: 547-558.

Ikezaki M, Kojima M, Sakakibara H, Kojima S, Ueno Y, Machida C, Machida Y (2010) Genetic networks regulated by ASYMMETRIC LEAVES1 (AS1) and AS2 in leaf development in Arabidopsis thaliana: KNOX genes control five morphological events. Plant J 61, 70–82

Kanrar S, Onguka O, Smith HMS (2006) Arabidopsis inflorescence architecture requires the activities of KNOX-BELL homeodomain heterodimers. Planta 224: 1163-1173.

Koornneef M, Vaneden J, Hanhart CJ, Stam P, Braaksma FJ, et al. (1983) Linkage map of *Arabidopsis thaliana*. J Hered 74: 265-272.

Venglat SP, Dumonceaux T, Rozwadowski K, Parnell L, Babic V, et al. (2002) The homeobox gene BREVIPEDICELLUS is a key regulator of inflorescence architecture in Arabidopsis. Proc Natl Acad Sci USA 99: 4730-4735.
