# Supplementary material for: Chromosome Fragile Sites in Arabidopsis Harbor Matrix Attachment Regions That May Be Associated with Ancestral Chromosome Rearrangement Events
Source: PLoS Genet. 2012 Dec 20;8(12):e1003136. doi: 10.1371/journal.pgen.1003136 (PMC3527283; doi:10.1371/journal.pgen.1003136)
Supplement: Table S2 — Primers used to localize deletion breakpoint regions. (PDF) [file pgen.1003136.s006.pdf]

Table S2: Primers used to localize deletions*

Name	Location	Sequence	Product size	C	L	1	2	3	5	11
F15N16 For	F15N16	CATGACCGAGAAGCCAAAGAGGAATCTC	0.35	+	+	+	+	+	+	+
F15N16 Back	83.3kb	CCTATTCTCTAACCGCAACTCTGCGTCG								
F14G16 For	F14G16	ACAAACCGATCAGCATTCAAG	0.25 (Col)	+	+	+	+	+	+	+
F14G16 Back	47.5kb	GCCTTTGTCACGGATTCAAC	0.2 (Ler)							
F28D6 For	F28D6	TGAAATGAACCAGTGCTGCTG	1.2	+	+	+	+	+	+	+
F28D6 Back	51.7kb	CTGTACCAATTAGTCGAGAAGGTC								
T3E15-2FOR	T3E15	GTAGCGTTATCCATGTGATAAGAGCGTCC	1.4 (Col)	+	+	+	+	+	+	-
T3E15-2BACK	49.4kb	CCTTTCCAATTTTGGCATGGTGAGAATTCAG	0.92 (Ler)							
T3E15 For	T3E15	GCCCATCAGTTTTCAAGCCCAAGAAGG	0.77	+	-	-	-	-	+	-
T3E15 back	116.9kb	CGCTACCTCTTCAAGTTCAACCATACGGAG								
F5K24 For	F5K24	CAGCGTCTAGGTCACCCATCTATGTCAAGG	1.75	+	-	-	-	-	+	-
F5K24 Back	38.2kb	CGGAGAACTTGTGAAACCATTGACGTGAAG								
F1K3 For	F1K3	GGGTACAAACATAGTCATTGGCAGG	1.7	+	+	-	-	+	+	-
F1K3 Back	23.8kb	AGACATTGAAATCTCCTCCTCGG								
F1K3-46 For	F1K3	GGTCCTTTGGTTTCTATGCGAAG	2.5	+	+	-	-	+	+	-
F1K3-47 Back	47.4kb	CTGAAAATTGTTCGTCGTCGGTAC								
44.7 For	T17A2	TTCCTCAACAGGGCTCACATTC	0.91	+	+	-	-	-	+	-
45.6 Back	1.4kb	GCAGGGATAAGAAAGGCATCTCC								
78.7 For	T13D4	CTGAAGCCCGAACCTAATGATG	0.74	+	+	-	+	-	+	-
79.4 Back	32.1kb	TGGTGGAGAATAGTTGCTGCG	<in Ler?							
76 For	F9M13	TGTGGATTTACTGGAACACTCCG	0.97 (Col)	+	+	-	-	-	+	-
77 Back	24.3kb	GCTCTGCCATCTTTTGACGAAG	Ler <							
North 8 For	F9M13	ACTCGGACATAGGCACTGTGACTC	0.97	+	+	-	-	-	-	-
North 8 Back	61.9kb	AACTCAGGAATGGAGAAGATGCG								
North 5 For	F9M13	ACCTTGGTTGCTTGGATGCC	2.0	+	+	-	-	-	-	-
North 5 Back	82.9kb	TGTCGCCGTTGTTAGTGTCTGAG								
BP GENE	F9M13 88kb									
bp2 FOR	T12G13	GAACATAAGGAGGGAATCATACCAAGAGG	2.3	+	+	-	+	-	-	-
bp2 BACK	2.7kb	GATGAAGCTAACGGACAACGAAGAGAT								
North 1 For	T12G13	GAGTCTTACCGATACAAAGGAGG	1.6	+	+	-	+	-	-	-
North 1 Back	11.3kb	AACTATCTGAACGCTTGTCCC								
T12G13 For	T12G13	GCACAATCGAGCAAGGTATGATG	1.85	+	+	-	+	-	-	-
T12G13 Back	20.5kb	ACGAGTCGAAGAAGCGTTTCGG								
T1223 For	T12G13	CGCTTAAAGGCTTGTACTTTCCCAC	2.25	+	+	-	+	-	-	-
T1223 Back	23.8kb	TGGCTAATCCTTGTTTGTGTGGAC								

South 3.25 For	T12G13	CCTCTCGTTTTTACGCCATTAGTCG	0.84	+	+	-	+	-	-	-
South 3.25 Back	29kb	TGAAGACGGATCTTGGATACTTAGGC								
South 3.75 For	T12G13	TTCCGAGATTTGCTTCGTCAATATTTTTAAGGG	0.57	+	+	-	+	-	-	-
South 3.75 Back	38.5kb	TAATCCAATCCCTAAATCCTAATTTGAGCTGT								
South 4 For	T12G13	TGAACTCCGTTGAGATAATGGGTG	0.85	+	+	+	+	-	-	-
South 4 Back	42.6kb	GGGACAGTCTTAGGCAATGGTGAC								
South 4.5 For	T12G13	GATCATCCCCGCTTATGAAAACGACATTC	0.62	+	+	+	+	-	-	-
South 4.5 Back	47.2kb	TGTCATTCCTTTGCCCGCACAATAGC								
bp10 For	T12G13	CGTTGGATCTCATAGATAGTAGAGTTATG	0.57	+	+	+	+	-	-	+
bp10 Back	51.8kb	TTGATAGATGTGCTTTTAAGTGAGTATCT								
South 4.75 For	T12G13	GCATCGCTTTTTACCATTGCTAAG	0.96	+	+	+	+	-	-	+
South 4.75 Back	60.6kb	GCCTGCTATTTCATTTATCAGTCTCG								
South 6 For	T12G13	TGTATCGTAATCTTGTGGCTGCTC	0.7	+	+	+	+	-	-	+
South 6 Back	77.2kb	ACGGTAAAGGAATAAGCGGACC								
South 6.25 For	T12G13	CCTCTGGATAACTGAAGCAGGCA	1.0	+	+	+	+	-	+	+
South 6.25 Back	80.8kb	GTGTCTCCTACACTCATGTTC								
08310 For	T12G13	GCTGAGCAAACAAAGGATGAGGAC	1.5	+	+	+	+	+	+	+
08310 Back	88.7kb	CTTCGTTTTGACTTCCCTCTCCG								
South 8 For	T28D6	TTGTAGGGGCAGAATCGCAAGC	0.8	+	+	+	+	+	+	+
South 8 back	5.4kb	GCAAGTCAATCAACGAATCAGGC								
South 9 For	T28D6	GCTGGAGAAAAGCGTAAGGAGGT	0.9	+	+	+	+	+	+	+
South 9 back	13.2kb	AGGGCAATGAGATGTGTTGGATAG								

*PCR conducted on genomic DNA from Columbia (C), Landsberg *erecta* (L), and the *bp* mutant alleles, *bp-1, bp-2, bp-3, bp-5* and *bp-11*. Product size is in kilobase pairs. The +/- symbols indicate the presence or absence of a pcr product with the given template/primer pair.
