# Supplementary material for: Chromosome Fragile Sites in Arabidopsis Harbor Matrix Attachment Regions That May Be Associated with Ancestral Chromosome Rearrangement Events
Source: PLoS Genet. 2012 Dec 20;8(12):e1003136. doi: 10.1371/journal.pgen.1003136 (PMC3527283; doi:10.1371/journal.pgen.1003136)
Supplement: Table S3 — iPCR primer sets. (PDF) [file pgen.1003136.s007.pdf]

Table S3: Inverse PCR primers*

Allele	Primer	Sequence
bp1	S4iPCR for2	CCTCTTTGGATACCGTGCTTGGGAG
	S4iPCR back	CCCTGGAGTACTAGCCTCAAAAGG
bp-2	bp2 iPCR for	CGTCAGTCAATGCTTCAGTGTGTTGCG
	bp2 iPCR back	CATGATATCTTGGATTCACATAGAAGTCATCCC
bp-3	XT1 iPCR for	CTGGCTATGGGAAGCGTGTTGA
	XT1 iPCR back	ACAACAGACTCTCCTCTCTGCCGG
bp-5	bp5 DB/back	TGCCTGCTTCAGTTATCCAGAGG
	South 6.25 back	GTGTCTCCTACACTCATGTTC
bp-11	bp11 iPCR For2	ACGGTCAAACGGTCAAAATACAAAAGGC
	bp11 iPCR Back	GCTCACTCATAATTTGAAATTGTTGTACCACC

*These primers pairs are in diverging orientations, enabling amplification of recircularized genomic DNA that had been resected with the RFLP enzyme.
