# Supplementary material for: Chromosome Fragile Sites in Arabidopsis Harbor Matrix Attachment Regions That May Be Associated with Ancestral Chromosome Rearrangement Events
Source: PLoS Genet. 2012 Dec 20;8(12):e1003136. doi: 10.1371/journal.pgen.1003136 (PMC3527283; doi:10.1371/journal.pgen.1003136)
Supplement: Table S4 — Phase 5 primer sets. (PDF) [file pgen.1003136.s008.pdf]

Table S4: Phase 5 primers*

| Allele | Name | Sequence | Product |
|---|---|---|---|
| *bp-3* | 22144bp3.seq2 | GTTCATAAAAGTGAGGGGGAGTAATG | 500bp |
| | BP3DB/B | CCTTCGTTTACTTCACTGG | |
| *bp-5* | 22145bp5.seq2 | AAGCACGAACAGTGGATCAAG | 770bp |
| | BP5DB/B | TGCCTGCTTCAGTTATCCAGAGG | |
| *bp-11* | *bp-11* phase 5 | CAGATAGGAGAGGAAGAAGCATCTACTCATC | |
| | *bp-11* iPCR FOR2 | GGTCAAACGGTCAAAATACAAAAGGC | 550bp |

*These primer sets amplify across the breakpoint junctions to generate PCR products of the indicated size.
