# Supplementary material for: Chromosome Fragile Sites in Arabidopsis Harbor Matrix Attachment Regions That May Be Associated with Ancestral Chromosome Rearrangement Events
Source: PLoS Genet. 2012 Dec 20;8(12):e1003136. doi: 10.1371/journal.pgen.1003136 (PMC3527283; doi:10.1371/journal.pgen.1003136)
Supplement: Table S5 — List of genes deleted in bp mutants. (PDF) [file pgen.1003136.s009.pdf]

Table S5: Annotations of genetic elements in the 4.29Mb–5.25Mb region encompassing *bp* deletions
Notes: North and south breakpoint locations of *bp1*, *bp2*, *bp3*, *bp5* and *bp11* are highlighted in green. Genes exhibiting evidence of expression (cDNA or EST clones) are highlighted in yellow, and GenBank accession numbers are given.

AGI ID	coordinate	annotation	cDNAs	ESTs	T-DNAs
	4287kb	*bp11* North breakpoint			
At4g07495	4289kb	transposable element gene. Mutator-like transposase family			SALK_138084
At4g07496	4293kb	transposable element gene; pseudogene,			
At4g07498	4297kb	transposable element gene; gypsy-like retrotransposon family (Athila)	BE529173		SALK_085566
At4g07500	4300kb	transposable element gene; similar to unknown protein			
At4g07502	4304kb	transposable element gene; gypsy-like retrotransposon family (Athila			GK-437B03
At4g07504	4307kb	transposable element gene; gypsy-like retrotransposon family (Athila			SAIL_382_G07
At4g07507	4310kb	transposable element gene; pseudogene, replication protein A1 -related	BX828000<br>AK227467	142L2T7<br>002203_0775_2321<br>RAFL14-52-L16 (3')	GK-718H02-025281<br>FLAG_544B01
At4g07510	4313kb	transposable element gene; similar to unknown protein			WiscDsLox421D5<br>GK-296D02-015495
At4g07515	4315kb	Protein of unknown function (DUF784)	AY089062<br>BT029248	079126_0374_3686	
At4g07516	4319kb	Transposable element gene; non-LTR retrotransposon family (LINE)			SALK_027830.44.00.x<br>FLAG_297D04<br>1 more
At4g07517	4323kb	transposable element gene; CACTA-like transposase family (Tnp2/En/Spm)			WiscDsLox241D07<br>GK-794F08-024939<br>2 more
At4g07518	4327kb	Transposable element gene; CACTA-like transposase family (Ptta/En/Spm)		AYAGJ24TF	GK-385A06-018252<br>GK-385A06-018248
At4g07519	4332kb	similarity to PttA-like transposon protein		CB25933	
At4g07521	4334kb	transposable element gene; similar to nucleic acid binding / zinc ion binding [Arabidopsis thaliana] (TAIR:AT2G01050.1)			GK-489H06-019644<br>GK-489H06-019637

At4g07522	4338kb	Transposable element gene; Mutator-like transposase family			SALK_064045.56.00.x
At4g07523	4341kb	Transposable element gene; similar to unknown protein	BT015491<br>BT014695		SALK_012605.29.99.f<br>SALK_012605.28.20.x.1<br>2 more
At4g07520	4344kb	transposable element gene; similar to unknown protein			GK-889G08-030257
At4g07524	4346kb	Ras-related small GTP-binding family protein			SALK_012605.54.70.x<br>GK-844B08-025742<br>1 more
At4g07525	4357kb	transposable element gene; gypsy-like retrotransposon family			GK-456C06-018779
At4g07526	4359kb	unknown protein		AYAWY10TF	SALK_003336.47.95.x<br>SALK_003332.44.80.x
At4g07528	4362kb	Transposable element gene; gypsy-like retrotransposon family			GK-048B06-016082
At4g07530	4367kb	Transposable element gene; similar to unknown protein		AYAXY54TR	SALK_117836.50.70.x<br>SALK_117827.45.95.x
At4g07540	4371kb	transposable element gene; hAT-like transposase family (hobo/Ac/Tam3)			GK-206D05-014513
At4g07550	4378kb	transposable element gene; gypsy-like retrotransposon family			
At4g07560	4381kb	Transposable element gene; transposon protein -related			GK-733F02-025442
At4g07563	4384kb	Transposable element gene; gypsy-like retrotransposon family (Athila)			
At4g07566	4387kb	transposable element gene; gypsy-like retrotransposon family (Athila)			
At4g07570	4390kb	Transposable element gene; pseudogene, hypothetical protein			SALK_060250<br>SALK_060250.55.25.x

At4g07580	4395kb	transposable element gene; pseudogene, Ulp1 protease family			
At4g07583	4398kb	transposable element gene; pseudogene, hypothetical protein			SAIL_1266_E05.v2
At4g07586	4405kb	Transposable element gene; pseudogene, hypothetical protein			
At4g07590	4407kb	transposable element gene; gypsy-like retrotransposon family			
At4g07595	4408kb	transposable element gene; pseudogene, hypothetical protein			
At4g07600	4412kb	transposable element gene; gypsy-like retrotransposon family (Athila)			
At4g07605	4416kb	Transposable element gene; pseudogene, hypothetical protein			SALK_111568.54.25.x
At4g07620	4420kb	transposable element gene; gypsy-like retrotransposon family (Athila)			SALK_104494.37.15.x<br>SALK_104477.19.20.x<br>3 more
At4g07630	4424kb	transposable element gene; pseudogene, hypothetical protein			SALK_015003.52.05.x<br>SALK_037396
At4g07640	4428kb	Transposable element gene; gypsy-like retrotransposon family (Athila)			SALK_015203.56.00.x<br>SALK_015203
At4g07650	4435kb	Transposable element gene; pseudogene, hypothetical protein			
At4g07660	4439kb	transposable element gene; gypsy-like retrotransposon family (Athila)			SALK_151280.23.30.x<br>GK-078C03-016176<br>5 more
At4g07662	4448kb	transposable element gene; pseudogene, helicase - related, similar to putative helicase			

At4g07664	4454kb	transposable element gene; gypsy-like retrotransposon family (Athila)			GK-241H01-014367 GK-433F04-018151 2 more
At4g07666	4458kb	unknown protein			SALK_150530.34.10.n
At4g07668	4462kb	transposable element gene; gypsy-like retrotransposon family			SALK_102493.50.85.x SALK_062200.37.80.x 4 more
At4g07670	4464kb	protease-associated (PA) domain-containing protein			SAIL_208_G01.v1
At4g07675	4467kb	unknown protein		AYAWY16TF	SALK_112044.44.00.x SALK_111948.45.90.x 2 more
At4g07680	4470kb	Transposable element gene; pseudogene, Ulp1 protease famiy			SAIL_813_A07.v1
At4g07685	4472kb	transposable element gene; pseudogene, hypothetical protein			
At4g07686	4474kb	transposable element gene; pseudogene, hypothetical protein			
At4g07688	4475kb	Transposable element gene; CACTA-like transposase family (Ptta/En/Spm)			
At4g07690	4477kb	Transposable element gene; similar to unknown protein			FLAG_431D08
At4g07693	4479kb	Transposable element gene; Mutator-like transposase family			
At4g07696	4483kb	transposable element gene; pseudogene, hypothetical protein			
At4g07699	4485kb	pseudogene of zinc knuckle (CCHC-type) family protein			
At4g07700	4486kb	Transposable element gene; gypsy-like retrotransposon family (Athila)			SALK_129316.21.60.n WiscDsLox393-396H24 1 more

At4g07703	4491kb	Transposable element gene; gypsy-like retrotransposon family (Athila)			
At4g07706	4496kb	transposable element gene; CACTA-like transposase family (En/Spm)			SAIL_798_B01.v1
At4g07713	4499kb	transposable element gene; Mutator-like transposase family			
At4g07720	4505kb	pseudogene, hypothetical protein			SALK_087497.51.9 5.x
At4g07725	4511kb	Transposable element gene; gypsy-like retrotransposon family			SALK_061828 SALK_061828.45.6 5.x 1 more
At4g07730	4516kb	transposable element gene; gypsy-like retrotransposon family (Athila)			GK-269B10-015062
At4g07733	4522kb	transposable element gene; gypsy-like retrotransposon family (Athila)			
At4g07736	4528kb	transposable element gene; gypsy-like retrotransposon family (Athila)			
At4g07738	4534kb	Transposable element gene; gypsy-like retrotransposon family (Athila)			GK-339E05-016160 SALK_018673.55.2 5.x
At4g07740	4540kb	Protein of unknown function (DUF3287)		AYALW80TR	
At4g07742	4542kb	Transposable element gene; gypsy-like retrotransposon family		AYALW80TF	SALK_146485.56.0 0.x
At4g07744	4546kb	transposable element gene; gypsy-like retrotransposon family			SALK_117101.46.0 0.x SALK_117099.31.2 5.n
At4g07747	4548kb	pseudogene, hypothetical protein		AYAIO63TR	
At4g07750	4553kb	Transposable element gene; CACTA-like transposase family (Tnp2/En/Spm)			FLAG_494H08 SALK_009814.12.4 5.x 3 more
At4g07755	4563kb	transposable element gene; gypsy-like retrotransposon family (Athila)			GK-157D07-013213 SALK_044319.51.5 5.x 4 more

At4g07760	4570kb	transposable element gene; CACTA-like transposase family (Tnp1/En/Spm)			
At4g07770	4594kb	transposable element gene; non-LTR retrotransposon family (LINE)			WiscDsLox433A1
At4g07780	4601kb	transposable element gene; gypsy-like retrotransposon family (Athila)			WiscDsLox288H07<br>SALK_022004.56.00.x<br>1 more
At4g07786	4605kb	This gene encodes a small protein and has either evidence of transcription or purifying selection			
At4g07790	4608kb	transposable element gene; hypothetical protein			
At4g07795	4612kb	transposable element gene; pseudogene, replication protein A1			GK-872E03-026119<br>GK-872E03-026084
At4g07800	4616kb	transposable element gene; similar to unknown protein			
At4g07803	4619kb	transposable element gene; pseudogene, helicase - related, similar to putative helicase			SALK_079650.53.10.x<br>SALK_079652.54.05.x
At4g07806	4626kb	Transposable element gene; copia-like retrotransposon family			GK-685E04-024533
At4g07810	4631kb	transposable element gene; copia-like retrotransposon family			SALK_136928.39.30.x<br>GK-053A05-012481<br>4 more
At4g07812	4635kb	unknown pseudogene			
At4g07820	4637kb	CAP (Cysteine-rich secretory proteins, Antigen 5, and Pathogenesis-related 1 protein) superfamily protein	S63135<br>U63135	178B9T7<br>EBENXNS01DHOID	FLAG_440H03<br>SAIL_902_D06.v1<br>3 more
At4g07825	4644kb	unknown protein	RAFL16-71-O16<br>GSLTLS24ZB04<br>8 more	RAFL16-71-O16 (5')<br>RAFL16-71-O16 (3')	GK-502C01-019715<br>GK-185F09-013650<br>6 more
At4g07830	4647kb	transposable element gene; gypsy-like retrotransposon family			SAIL_323_F10.v1

At4g07840	4654kb	Transposable element gene; copia-like retrotransposon family	AY299292		SALK_018389.56.0 0.x SALK_141819.24.9 5.x
At4g07841	4661kb	Pseudogene of AT1G24440; protein binding / zinc ion binding protein			
At4g0743	4666kb	Transposable element gene; hAT-like transposase family (hobo/Ac/Tam3)			
At4g0746	4670kb	transposable element gene; gypsy-like retrotransposon family			
At4g07850	4673kb	transposable element gene; gypsy-like retrotransposon family	BT011813	AYAVM63TR AYAVM63TF	GK-042G09-011515 SALK_084701.36.6 5.x
At4g07856	4681kb	transposable element gene; gypsy-like retrotransposon family			SALK_044669.17.3 5.x WiscDsLox317H05
At4g07862	4688kb	transposable element gene; copia-like retrotransposon family			SALK_011297.43.5 0.x
At4g07868	4692kb	unknown protein	AY735639	AYALX06TR AYALW93TF	GK-301F06-015558
At4g07869	4693kb	Pseudogene of AT1G24440; protein binding / zinc ion binding protein			
At4g07874	4698kb	transposable element gene; hAT-like transposase family (hobo/Ac/Tam3)			SALK_104937.41.2 5.x SALK_104938.51.8 0.x 3 more
At4g07890	4705kb	transposable element gene; gypsy-like retrotransposon family (Athila)			SALK_090624.47.2 0.x
At4g07893	4707kb	transposable element gene; pseudogene, hypothetical protein, similar to contains similarity to Arabidopsis thaliana hypothetical protein (GB:AC004483)			SALK_024257.30.6 5.x SALK_129300.24.3 0.n 3 more
At4g07896	4709kb	Transposable element gene; pseudogene, hypothetical protein, similar to contains similarity to Arabidopsis thaliana hypothetical protein (GB:AC004483)			SALK_152925.36.2 0.x GK-820E02-025640 1 more

At4g07915	4718kb	Transposable element gene; non-LTR retrotransposon family (LINE)			SALK_066772<br>SALK_066772.51.7<br>5.x<br>2 more
At4g07917	4722kb	Transposable element gene; gypsy-like retrotransposon family			
At4g07920	4725kb	Transposable element gene; pseudogene, hypothetical protein			GK-640B04-<br>022275<br>SALK_046111.54.2<br>5.x
At4g07931	4729kb	transposable element gene; gypsy-like retrotransposon family			SALK_135546.26.4<br>0.x<br>SALK_135547.48.1<br>5.x<br>1 more
At4g07932	4733kb	unknown protein		AYAXX31TR	GK-888E05-<br>030256<br>SAIL_174_G07.v1
At4g07933	4735kb	transposable element gene; gypsy-like retrotransposon family			
At4g07934	4740kb	Transposable element gene; gypsy-like retrotransposon family (Athila)			
At4g07935	4746kb	transposable element gene; gypsy-like retrotransposon family			SALK_141832.40.6<br>0.x<br>SALK_104300.41.5<br>5.x<br>9 more
At4g07946	4751kb	transposable element gene; CACTA-like transposase family (Ptta/En/Spm)			
At4g07947	4754kb	Transposable element gene; pseudogene, hypothetical protein			SALK_147424.42.4<br>0.x<br>SALK_147432.38.7<br>0.x<br>6 more
At4g07936	4757kb	transposable element gene; pseudogene, hypothetical protein			SALK_065597<br>SALK_129254.44.4<br>5.x<br>5 more

At4g07937	4760kb	transposable element gene; gypsy-like retrotransposon family (Athila)			SALK_126523.34.4 5.x<br>GK-209E09-014556<br>1 more
At4g07938	4766kb	Transposable element gene; gypsy-like retrotransposon family (Athila)			GK-110F06-030270<br>GK-438C03-018206<br>1 more
At4g07939	4769kb	Transposable element gene; pseudogene, hypothetical protein, similar to contains similarity to Arabidopsis thaliana hypothetical protein (GB:AC004483)			SALK_059927.51.0 5.x
At4g07941	4772kb	transposable element gene; gypsy-like retrotransposon family (Athila)			
At4g07942	4775kb	transposable element gene; pseudogene, hypothetical protein, similar to Athila retroelement ORF2, putative			
At4g07943	4784kb	Transposable element gene; similar to unknown protein		AYAZD05TF	
At4g07944	4785kb	Transposable element gene; similar to unknown protein		AYALX20TF	
At4g07945	4789kb	transposable element gene; pseudogene, similar to putative reverse transcriptase, similar to reverse transcriptase, putative			
At4g07940	4793kb	Protein of unknown function (DUF3245)			SALK_091810.49.0 0.x<br>FLAG_347D05<br>7 more
At4g07950	4799kb	DNA-directed RNA polymerase, subunit M, archaeal	RAFL09-96-C05<br>U25511	17D02<br>701495736	GK-412F10-018001<br>SAIL_823_B06.v3<br>4 more
At4g07960	4804kb	encodes a gene similar to cellulose synthase	RAFL19-71-L23<br>U60875	RAFL19-32-H22 (3')<br>600037971R1 (5')<br>3 more	SAIL_168_F02<br>SALK_096810<br>9 more
At4g07965	4809kb	unknown protein		AYALX36TF	FLAG_526B04<br>SALK_137099.18.2 0.x<br>1 more

At4g07967	4813kb	transposable element gene; pseudogene, hypothetical protein			SALK_001679.55.5<br>0.x<br>SAIL_878_H09<br>8 more
At4g07970	4818 kb	Transposable element gene; hypothetical protein, similar to A. thaliana hypothetical proteins			SALK_040916.28.2<br>0.n
At4g07990	4828kb	Chaperone DnaJ-domain superfamily protein	R11662<br>U11662	RAFL19-33-G09 (3')<br>02L02 (3')<br>2 more	SALK_018033<br>SALK_086123.38.8<br>0.n<br>4 more
At4g08000	4832kb	Transposable element gene; CACTA-like transposase family (Tnp2/En/Spm)			FLAG_163C11
At4g08010	4834kb	Transposable element gene; CACTA-like transposase family (En/Spm)			WiscDsLox457-<br>460K5
At4g08013	4836kb	transposable element gene; similar to unknown protein		AYALX44TF	
At4g08016	4838kb	transposable element gene; CACTA-like transposase family (Ptta/En/Spm)		AYALX44TF<br>AYALX40TR	
At4g08020	4840kb	transposable element gene; CACTA-like transposase family (En/Spm)			FLAG_249C11<br>FLAG_249C10
At4g08022	4845kb	pseudogene, hypothetical protein		062174_1642_3264<br>083931_1338_0700	
At4g08025	4847kb	Protein of unknown function (DUF784)		AYBID02TR	SALK_149463.45.2<br>5.x<br>SALK_044554.42.5<br>5.x
At4g08028	4852kb	Encodes a defensin-like (DEFL) family protein.			
At4g08030	4856kb	transposable element gene; gypsy-like retrotransposon family (Athila)			
	4861kb	*bp3* north breakpoint			
At4g08032	4864kb	transposable element gene; pseudogene, similar to putative transposable element			GK-228E05-<br>014267
At4g08035	4867kb	Unknown gene	GSLTSIL37ZE05<br>RAFL25-48-G05<br>3 more	RAFL25-48-G05 (5')<br>701554386<br>3 more	SALK_019771.39.9<br>5.x<br>SALK_066519.56.0<br>0.x<br>5 more

At4g08033	4877kb	transposable element gene; pseudogene, hypothetical protein			GK-623G04-022313 GK-634B04-022366 2 more
At4g08034	4879kb	transposable element gene; pseudogene, similar to putative non-LTR retroelement reverse transcriptase, similar to reverse transcriptase, putative			
At4g08036	4880kb	transposable element gene; non-LTR retrotransposon family (LINE)			
At4g08038	4881kb	Transposable element gene; pseudogene, hypothetical protein, similar to reverse transcriptase, putative			
At4g08039	4885kb	Encodes a defensin-like (DEFL) family protein.			GK-444E12-024528 GK-444F12-024528 2 more
At4g08040	4888kb	Encodes an aminotransferase that belongs to ACC synthase gene family structurally	C00055 (E)		GK-284B12-015276 GK-284B12-015289 1 more
At4g08050	4899kb	transposable element gene; gypsy-like retrotransposon family (Athila)			SALK_073847.50.75.x SALK_151095.51.05.x
At4g08053	4913kb	transposable element gene; CACTA-like transposase family (En/Spm)			
At4g08054	4918kb	transposable element gene; copia-like retrotransposon family			SALK_059626.19.85.x
At4g08056	4922kb	transposable element gene; similar to unknown protein			
At4g08060	4927kb	transposable element gene; CACTA-like transposase family (Tnp1/En/Spm)		PI32	SALK_018653.36.75.x SAIL_900_C11.v2 2 more
At4g08070	4930kb	Transposable element gene; CACTA-like transposase family (Tnp2/En/Spm)			GK-447A03-024539 GK-447A03-024530 3 more
At4g08071	4934kb	pseudogene, hypothetical protein			

At4g08072	4946kb	transposable element gene; gypsy-like retrotransposon family (Athila)			
At4g08073	4950kb	This gene encodes a small protein and has either evidence of transcription or purifying selection.			
At4g08074	4950kb	Transposable element gene; gypsy-like retrotransposon family			
At4g08076	4953kb	transposable element gene; gypsy-like retrotransposon family (Athila)			
At4g08078	4962kb	transposable element gene; gypsy-like retrotransposon family (Athila)			SALK_110250.18.85.n SAIL_1187_H11.v1 1 more
At4g08080	4965kb	transposable element gene; gypsy-like retrotransposon family (Athila)			SALK_024043.35.20.x SALK_079126.42.95.x 1 more
At4g08090	4974kb	transposable element gene; CACTA-like transposase family (Ptta/En/Spm)			GK-272C05-015096 GK-854A10-025755 8 more
At4g08091	4982kb	transposable element gene; CACTA-like transposase family (Ptta/En/Spm)		AYARA33TR AYALX59TF 1 more	SALK_073643.24.45.x
At4g08092	4987kb	transposable element gene; CACTA-like transposase family (Tnp2/En/Spm)			SALK_048617.52.35.x SAIL_1221_A08.v1
At4g08093	4991kb	pseudogene of unknown protein		M20D2 (5') 271717 (5')	SALK_026461.35.40.x
At4g08094	4994kb	Transposable element gene; copia-like retrotransposon family			SALK_031378.49.75.x SALK_143010.41.55.x
At4g08095	5000kb	transposable element gene; gypsy-like retrotransposon family			SALK_074931.41.85.x SALK_125636.40.65.x
At4g08096	5002kb	Transposable element gene; gypsy-like retrotransposon family			
At4g08097	5005kb	BEST Arabidopsis thaliana protein match is: myosin heavy chain-related (TAIR:AT3G30230.1)			
At4g08098	5007kb	pseudogene of hypothetical protein			

At4g08099	5010kb	transposable element gene; gypsy-like retrotransposon family			GK-137F04-012753<br>GK-137F04-012802<br>1 more
At4g08101	5013kb	Transposable element gene; gypsy-like retrotransposon family (Athila)			WiscDsLox367D1_010<br>GK-138E10-012752<br>2 more
At4g08102	5016kb	Transposable element gene; gypsy-like retrotransposon family			
At4g08103	5019kb	Transposable element gene; gypsy-like retrotransposon family (Athila)			SALK_125533.42.60.x
At4g08116	5020kb	Encodes a microRNA			
At4g08104	5022kb	transposable element gene; gypsy-like retrotransposon family (Athila)			SALK_091129.54.50.x
At4g08105	5025kb	Transposable element gene; gypsy-like retrotransposon family	RAFL22-59-I21		
At4g08106	5028kb	transposable element gene; gypsy-like retrotransposon family (Athila)	RAFL22-59-I21		
At4g08107	5032 kb	Transposable element gene; gypsy-like retrotransposon family			
At4g08108	5042 kb	transposable element gene; copia-like retrotransposon family			GK-333E06-016060<br>GK-018A02-013490<br>1 more
At4g08109	5052kb	Transposable element gene; gypsy-like retrotransposon family (Athila)			SAIL_326_C03.v1<br>SAIL_326b_C03.v1<br>3 more
At4g08111	5056kb	transposable element gene; similar to unknown protein			SALK_020553.54.50.x
At4g08112	5062kb	Transposable element gene; gypsy-like retrotransposon family (Athila)			GK-605F11-021294<br>GK-605F11-021275<br>1 more
At4g08113	5066kb	transposable element gene; similar to unknown protein			GK-389A09-017791<br>GK-389A09-018261

At4g08114	5071kb	transposable element gene; gypsy-like retrotransposon family			FLAG_387C08<br>GK-476E06-019131<br>1 more
At4g08115	5077kb	Transposable element gene; gypsy-like retrotransposon family		AYBCC39TF<br>APZL23H09F (3')<br>1 more	SALK_093738<br>SALK_103945.23.15.x<br>4 more
At4g08100	5082kb	transposable element gene; gypsy-like retrotransposon family			SALK_146037.37.75.x<br>SAIL_1293_A10.v1<br>4 more
At4g08110	5085kb	transposable element gene; CACTA-like transposase family (Ptta/En/Spm)	RAFL16-08-P11<br>GSLTLS87ZB03<br>3 more	RAFL16-08-P11 (3')<br>FAFM91<br>2 more	SALK_129167.17.95.x<br>GK-903B01-030004<br>6 more
At4g08120	5091kb	transposable element gene; CACTA-like transposase family (Tnp1/En/Spm)			SALK_043486.38.45.x<br>GK-872E07-026084<br>1 more
At4g08130	5094kb	transposable element gene; similar to unknown protein			
	5101kb	*bp5* north breakpoint			
At4g08131	5104kb	transposable element gene; gypsy-like retrotransposon family (Athila)			GK-004D03-014797<br>FLAG_415F10
At4g08132	5107kb	transposable element gene; gypsy-like retrotransposon family (Athila)			GK-544F05-020964<br>FLAG_589F03<br>2 more
At4g08134	5117kb	transposable element gene; gypsy-like retrotransposon family			GK-079H05-011917<br>GK-708C09-022874<br>5 more
At4g08135	5120kb	Transposable element gene; gypsy-like retrotransposon family (Athila)			GK-270H02-015063<br>SALK_071610.56.00.x<br>3 more

At4g08136	5124kb	purple acid phosphatase. putative, similar to purple acid phosphatase (GI:20257485)			GK-440E11-018208<br>GK-438E11-018206<br>1 more
At4g08138	5131kb	transposable element gene; gypsy-like retrotransposon family			GK-335B02-016062<br>GK-151A11-013138<br>4 more
At4g08140	5136kb	BEST Arabidopsis thaliana protein match is: 26S proteasome regulatory subunit S2 1A (TAIR:AT2G20580.1)			AY202294<br>AY027345<br>3 more
At4g08145	5139kb	transposable element gene; hypothetical protein, contains Pfam domain, PF04827: Protein of unknown function (DUF635)			SAIL_1245_C08.v1
At4g08150	5149kb	*BREVIPEDICELLUS*: A member of class I knotted1-like homeobox gene family (together with KNAT2)	R10690<br>U10690<br>2 more	EB3RODY02GUHBF<br>EB3RODY01CBBQK<br>2 more	SALK_137958.48.30.x<br>SAIL_119_C02<br>1 more
At4g08151	5152kb	This gene encodes a small protein and has either evidence of transcription or purifying selection			
At4g08160	5161kb	Encodes a putative glycosyl hydrolase family 10 protein (xylanase)	RAFL16-91-G20<br>GSLTPGH76ZB12<br>2 more	RAFL16-91-G20 (3')<br>RAFL16-91-G20 (5')	GK-692G06-025071<br>SAIL_1290_G04<br>4 more
	5164kb	*bp2* south breakpoint			
At4g08170	5165kb	Inositol 1,3,4-trisphosphate 5/6-kinase family protein	GSLTPGH47ZH05<br>R13178<br>4 more	RAFL07-60-I09 (3')<br>E11A5T7<br>4 more	ET7116.Ds5.03.28.01.JU61.b.361<br>SALK_120653.40.80.x<br>6 more
At4g08176	5168kb	This gene encodes a small protein and has either evidence of transcription or purifying selection.			SALK_021382.43.45.x<br>FLAG_267D07
At4g08180	5171kb	OSBP(oxysterol binding protein)-related protein 1C (ORP1C)	R22195<br>GSLTFB25ZC08<br>3 more	RAFL07-18-E16 (5')<br>RAFL23-07-B20 (5')<br>8 more	SALK_089877.20.05.x<br>SALK_040837<br>4 more

At4g08190	5175kb	P-loop containing nucleoside triphosphate hydrolases superfamily protein			SALK_019066.31.0 0.x
At4g08200	5182kb	Transposable element gene; similar to unknown protein			GK-157D07-013241
At4g08210	5185kb	Pentatricopeptide repeat (PPR-like) superfamily protein		160195_0420_1813	SAIL_90_H03.v1
At4g08220	5187kb	transposable element gene; Mutator-like transposase family		MPIZP2001G133Q (5-PRIME)<br>171747_2172_1710	SALK_049189.50.9 0.x<br>GK-299F09-015556<br>7 more
At4g08230	5189kb	glycine-rich protein	GSLTSIL53ZD10<br>RAFL09-43-M03<br>1 more	RAFL21-95-G10 (5')	SALK_021428.51.4 5.n<br>SALK_013775<br>10 more
At4g08240	5196kb	unknown protein	RAFL21-47-N24<br>GSLTSIL36ZE09<br>2 more	EBENXNS02IXFOZ<br>EBENXNS01DDT7D	SALK_010682.53.7 5.x<br>WiscDsLox289_29 2M12<br>1 more
At4g08250	5197kb	GRAS family transcription factor	BT011797	044663_3632_2523<br>EB3RODY01BLU4B<br>2 more	CT955475<br>CT955474<br>2 more
At4g08260	5201kb	Protein phosphatase 2C family protein			
	5203kb	*bp1* south breakpoint			
At4g08262	5207kb	Transposable element gene; non-LTR retrotransposon family (LINE)			SAIL_1258_A09.v1<br>SALK_101212<br>8 more
	5212kb	*bp11* south breakpoint			
At4g08263	5212kb	BEST Arabidopsis thaliana protein match is: DNA-binding storekeeper protein-related transcriptional regulator (TAIR:AT4G00610.1)			GK-646B03-023221<br>GK-563F06-022810<br>1 more
At4g08264	5215kb	transposable element gene; pseudogene, similar to putative transposable element, similar to transposase, putative			SALK_144748.52.2 5.x<br>SALK_017228.55.5 0.x

					7 more
At4g08267	5217kb	hAT transposon superfamily protein			SALK_132411.37.8 5.x
At4g08270	5218kb	BEST Arabidopsis thaliana protein match is: DNA binding (TAIR:AT3G47680.1)			
At4g08275	5223kb	Transposable element gene; gypsy-like retrotransposon family (Athila)			
At4g08280	5231kb	Thioredoxin superfamily protein	R12554<br>U12554	EB3RODY01A7BRW<br>EB3RODY02JJPL7<br>1 more	SALK_046593.53.1 5.x<br>SALK_076766.50.0 0.x<br>3 more
At4g08290	5240kb	nodulin MtN21 /EamA-like transporter family protein	RAFL19-85-H13<br>GSLTLS26ZA04<br>2 more	RAFL19-85-H13 (5')<br>701500489<br>2 more	SALK_076704.39.8 5.x<br>SAIL_448_D06.v1<br>5 more
	5242kb	*bp5* south breakpoint			
At4g08300	5246kb	nodulin MtN21 /EamA-like transporter family protein	RAFL16-91-H07<br>GSLTFB90ZD06<br>1 more	RAFL16-91-H07 (5')<br>EBENXNS02G6CXN<br>3 more	SAIL_686_A11.v1
	5248kb	*bp3* south breakpoint			
At4g08310	5250kb	BEST Arabidopsis thaliana protein match is: unknown protein (TAIR:AT1G44780.2)*	R09713<br>U09713<br>1 more	RAFL21-11-O10 (3')<br>EB3RODY01AW271<br>5 more	GK-413F08-017998<br>FLAG_376F07<br>7 more
