# Supplementary material for: Chromosome Fragile Sites in Arabidopsis Harbor Matrix Attachment Regions That May Be Associated with Ancestral Chromosome Rearrangement Events
Source: PLoS Genet. 2012 Dec 20;8(12):e1003136. doi: 10.1371/journal.pgen.1003136 (PMC3527283; doi:10.1371/journal.pgen.1003136)
Supplement: Table S6 — Primers used to generate southwestern blot probes. (PDF) [file pgen.1003136.s010.pdf]

Table S6: Primers used to generate southwestern blot probes*

Name	Sequence
PC MAR For	TTTGTGTTGTAAGGTGACACTAATGAATTG
PC MAR Back	GAGTATTGACATGCATTGGTAAGCG
bp-2 South For	GGCAATGATAACCACTAAAATCTCATACC
bp-2 South Back	CTTCCAGAGGCATAGAACTGTAACCTC
bp-3 North For	TCCTGAAGCAAACAATAGAGTAAATCAAG
bp-3 North Back	ACCTAGTAAAAACATAGTAGAAGTGGGCG
bp-3 South For	TATCGGTGGAAGAGAAAATAGGAATGC
bp-3 South Back	TTGGTTGCAGAGGATGGTGGTGAAG
bp-5 North For	TGTCGCCGTTGTTAGTGTCTGAG
bp-5 North Back	ACCTTGGTTGCTTGGATGCC
bp-11 South For	CGTTGGATCTCATAGATAGTAGAGTTATG
bp-11 South Back	TTGATAGATGTGCTTTTAAGTGAGTATCT
Histone H1 (At1g06760) For	CTTCACAATCCTCATAATCACTTTCG
Histone H1 (At1g06760) Back	GGTAGTCCAAAGGAACTGTGAATAAC

*These primers were used to generate PCR fragments that were subsequently end labeled to produce radiolabelled probes for southwestern blotting.
